# Synthesis, X-ray Analysis, Biological Evaluation and Molecular Docking Study of New Thiazoline Derivatives

**DOI:** 10.3390/molecules24091654

**Published:** 2019-04-26

**Authors:** Yahia N. Mabkhot, H. Algarni, Abdulrhman Alsayari, Abdullatif Bin Muhsinah, Nabila A. Kheder, Zainab M. Almarhoon, Faiz A. Al-aizari

**Affiliations:** 1 Department of Pharmaceutical Chemistry, College of Pharmacy, King Khalid University, Abha 61441, Saudi Arabia; 2 Department of Physics, Faculty of Sciences, King Khalid University, P.O. Box 9004, Abha 61441, Saudi Arabia; halgarni@kku.edu.sa; 3 Research Centre for Advanced Materials Science (RCAMS), King Khalid University, P.O. Box 9004, Abha 61441, Saudi Arabia; 4 Department of Pharmacognosy, College of Pharmacy, King Khalid University, Abha 61441, Saudi Arabia; alsayari@kku.edu.sa (A.A.); ajmohsnah@kku.edu.sa (A.B.M.); 5 Department of Chemistry, Faculty of Science, Cairo University, Giza 12613, Egypt; nabila.abdelshafy@gmail.com; 6 Department of Chemistry, College of Science, King Saud University, P.O. Box 2455, Riyadh 11451, Saudi Arabia; zalmarhoon@ksu.edu.sa (Z.M.A.); faizalaizari@yahoo.com (F.A.A.-a.); 7 Department of Chemistry, Faculty of Science, AL-Baydha University, Albaydah 38018, Yemen

**Keywords:** thiazoline, X-ray crystallography, molecular docking, antimicrobial activity, cytotoxic activity

## Abstract

A series of new thiazoline derivatives were synthesized. Structure analyses were accomplished employing ^1^H-NMR, ^13^C-NMR, X-ray and MS techniques. The in vitro antitumor activities were assessed against human hepatocellular carcinoma (HepG-2) and colorectal carcinoma (HCT-116) cell lines. The results revealed that the thiazolines **5b** and **2c** exhibited significant activity against the two cell lines. The in vitro antimicrobial screening showed that the thiazolines **2c**, **5b** and **5d** showed promising inhibition activity against *Salmonella* sp. Additionally, the inhibition activity of thiazolines **2e** and **5b** against *Escherichia coli* was comparable to that of the reference compound gentamycin.

## 1. Introduction

Thiazoline-based compounds possess a variety of biological activities such as antiproliferative [1,2,3,4,5,6,7,8,9,10], anti-inflammatory [11], antimicrobial, [12] and antioxidant properties [13]. In addition, they are reported as butyrylcholinesterase and carboxylesterase inhibitors [14]. Motivated by the above-mentioned results, numerous design and synthesis efforts have been employed to develop new derivatives with more effective and safer therapeutic profiles [15,16,17,18,19,20,21,22,23,24,25,26,27,28,29,30]. Additionally, heterocycles based on a thiazoline-2-thione core may undergo several chemical reactions, including alkylation, oxidation, and cycloaddition, as a result of having two different nitrogenous and sulfurous groups [31,32,33,34,35,36,37]. There are many methods for preparing thiazoline-2-thione derivatives from alkyl ammonium dithiocarbamates with the appropriate α-halocompound in the presence of an acid [38,39,40,41,42] or by using primary amines, CS_2_ and α-haloketone in DMF [43] or toluene [44] or in water as a solvent [45], or without solvent or catalyst [46]. These findings encouraged us to conduct a slightly modification of the reported reaction condition [45,47,48] to synthesize new thiazoline derivatives and to investigate their synthetic potential in the preparation of new thiazoline based heterocycles in order to assess their biological activities. 

## 2. Results and Discussion

### 2.1. Chemistry

**Scheme 1:**
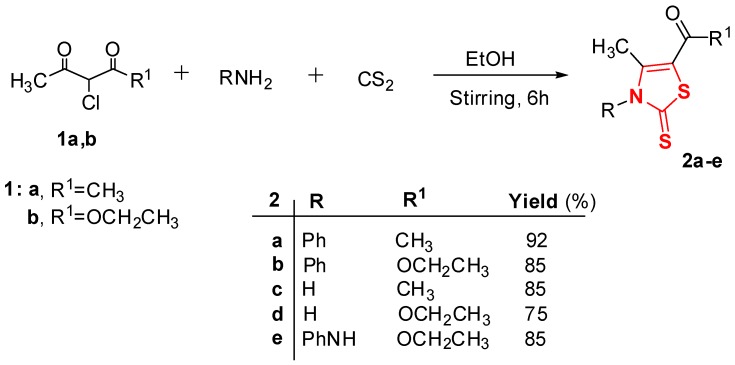
Synthesis of the target thiazoline-2-thiones **2a**–**e**.

The first series consisted of stirring 3-chloropentane-2,4-dione (**1a**) or ethyl 2-chloro-3-oxobutanoate (**1b**) with the appropriate primary amine and carbon disulfide in ethanol at room temperature; this afforded the target thiazoline-2-thione derivatives **2a**–**e** in high yields (Scheme 1).

**Scheme 2:**
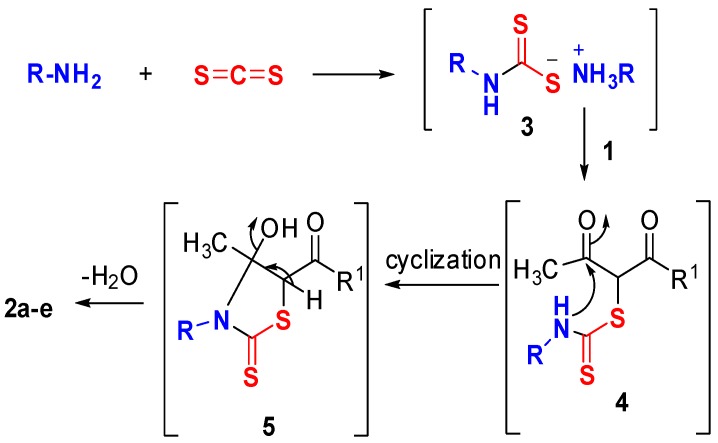
A suggested mechanism for the synthesis of target compounds **2a**–**e**.

The suggested mechanism for their synthesis is illustrated in Scheme 2. In this mechanism, the treatment of aniline with carbon disulfide results ammonium dithiocarbamate **3**, which reacts with α-halo-1,3-diketone **1** to produce acyclic dithiocarbamate derivative **4**. Intramolecular cyclization of intermediate **4**, followed by dehydration, yields the target compounds **2a**–**e** (Scheme 2).

The structures of the obtained products **2a**–**e** were established and confirmed via spectroscopic (NMR, MS, IR) and elemental analyses. Their ^1^H-NMR spectra showed the presence of a singlet signal due to methyl protons at carbon 4, in addition to the other expected proton signals. In addition, their ^13^C-NMR spectra confirmed the assigned structures and revealed the presence of the expected signals of carbonyl and thiocarbonyl at δ 177.5–188.17 and δ 190.0–193.18, respectively (see the Materials and Methods section). It is important to note that compounds **2a**–**d** were prepared by another two methods as follows: (i) in 54% and 74% yield, respectively, by using the same reagents as for compounds **2a**,**b** in the presence of NaOH as basic catalyst [47]; (ii) in yields of 74% and 75%, respectively, by the second literature method for synthesis of derivatives **2c**,**d** from α-halo-compounds with ammonium dithiocarbamate [48]. Our method for synthesis of compounds **2a**–**e** features better yields of these compounds. 

**Scheme 3:**
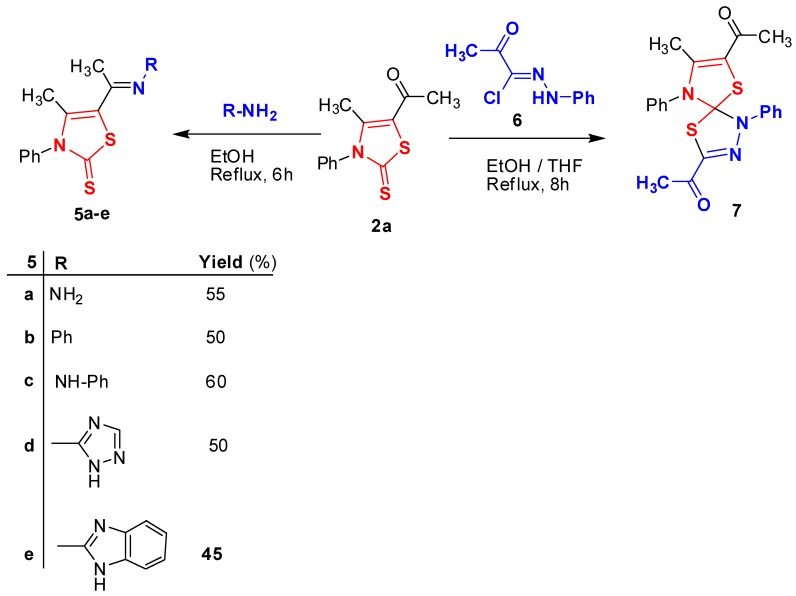
Synthesis of thiazoline drivatives **5a**–**e**, **7**, and **8**.

**Figure 1:**
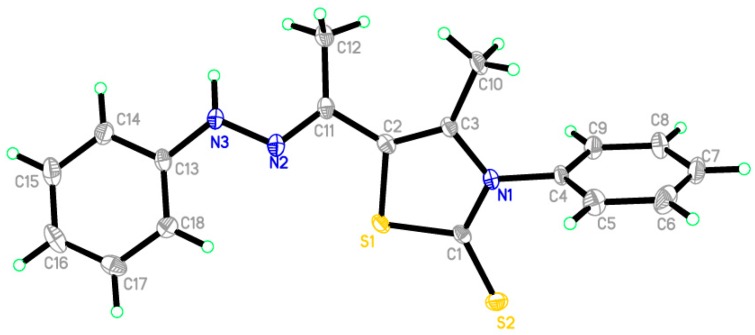
ORTEP diagram of the thiazoline **5c**. Displacement ellipsoids are plotted at the 40% probability level for non-H atoms.

Refluxing compound **2a** with the appropriate aniline derivatives afforded a new series of thiazoline derivatives, **5a**–**e**, as shown in Scheme 3. The structures of the target products **5a**–**e** were deduced from their IR, NMR and mass spectra. For example, their IR spectra revealed the absence of any absorption band due to the carbonyl group, which was apparent in compound **2a**. Also, the ^13^C-NMR spectra of the synthesized products **5a**–**e** showed, in each case, the absence of the carbonyl signal (see the Materials and Methods section). Moreover, the structure of **5c**, as a typical example of the prepared series, was confirmed by single crystal X-ray analysis (Figure 1). 

Next, the reaction of the thiazoline-2-thione derivative **2a** [45,47] with 2-oxo-*N*′-phenylpropane hydrazonoyl chloride (**6**) [49,50] produced the spiro-compound **7 [51]** (Scheme 3). The reaction was assumed to proceed via a 1,3-dipolar cycloaddtion reaction between nitrile imine (formed in situ from hydrazonoyl halide **6** by the action of triethylamine) and C=S of thiazoline-2-thione derivative **2a**. The ^1^H-NMR spectrum of spiro-compound **7** presented three signals at δ 1.92, 2.10 and 2.22, corresponding to three sets of methyl group protons. Additionally, its spectrum showed signals of the phenyl ring in the 7.35–7.59 ppm region. Also, the signal of C=S disappeared in its ^13^C-NMR spectrum.

### 2.2. X-ray Analysis

**Figure 2:**
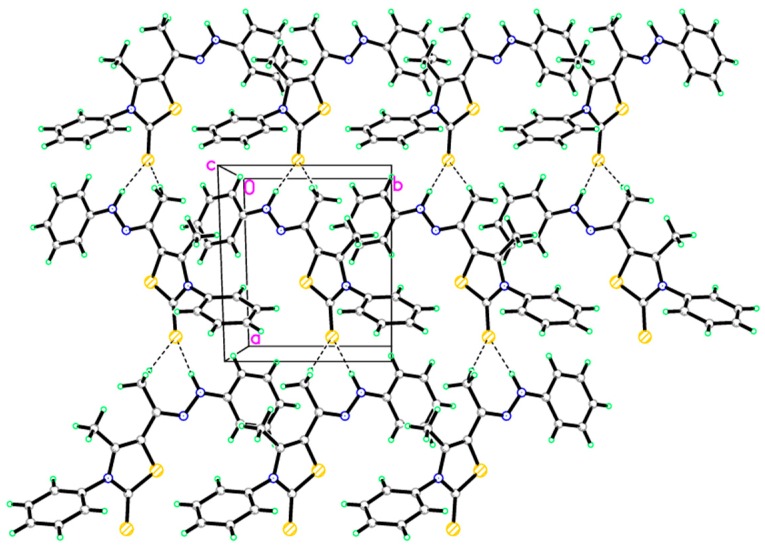
Molecular packing of thiazoline **5c** viewed hydrogen bonds, which are drawn as dashed lines along a axis.

The crystallographic data of thiazoline derivative **5c** and the refinement information are summarized in Table 1. The selected bond lengths and bond angles are listed in Table 2. The asymmetric unit contains one independent molecule, as shown in Figure 1. All the bond lengths and angles are in normal ranges [52]. In the crystal packing, shown in Figure 2, molecules are linked via one intermolecular hydrogen bond (Table 3).

The Crystallographic data for thiazoline **5c** (CCDC Number 1818811) can be obtained on request from the director, Cambridge Crystallographic Data Center, 12 Union Road, Cambridge CB2 1EW, UK.

### 2.3. Biological Evaluation

#### 2.3.1. The In Vitro Antimicrobial Assessment of the Synthesized Thiazolines

The antifungal and antibacterial potency of the synthesized compounds and the reference drugs were evaluated against two fungal species (*Aspergillus fumigatus* (RCMB 002008 (4) and *Candida albicans* (RCMB 05036)), two gram positive bacteria *(Staphylococcus aureus* (RCMB010010) and *Bacillus subtilis* (RCMB 010067)), and two gram negative bacteria (*Salmonella* sp. (RCMB 010043) and *Escherichia coli* (RCMB 010052)) using the inhibition zone technique according to the reported methods [53,54]. The results of this assessment are depicted in Table 4. Tests indicate that the compounds **2c**, **2d**, **2e**, and **5d** had significant antifungal activity against *Aspergillus fumigatus*. Additionally, all the evaluated thiazolines, except **2b**, were effective against *Candida albicans*. The study also showed that the tested compounds had important biological effectiveness against *Staphylococcus aureus* and *Bacillus subtilis* (except **2b**). The evaluation results showed that all the test compounds were effective against *Salmonella* sp., particularly **2c**, **5b** and **5d**; they approach the potency of Gentamycin. Moreover, the inhibition potency of thiazolines **2e** and **5b** is similar to the potency of Gentamycin towards *Escherichia coli*.

#### 2.3.2. Molecular Docking

The docking study play important role in predicting with the biological activity of any compounds. So, this study is considered a key for design and manufacturing of new drugs. We selected two derivatives **2c** and **5b** from the two synthesized series to use in molecular docking to study their behavior and their mode of action. Both thiazoline derivatives **2c** and **5b** were docked with human cyclin-dependent kinase enzyme (CDK 2), one of the kinase family, due to their important role in cell meiosis and replication.

Molecular docking was implemented using the MOE 2014.010 Package software. The structure of CDK2 was obtained from a protein data bank. Protein was optimized by adding hydrogen, repairing broken amino acid residues and removing water. In addition, compounds **2c** and **5b** were optimized for docking by adding hydrogen and then forcing energy minimization. 

**Figure 3:**
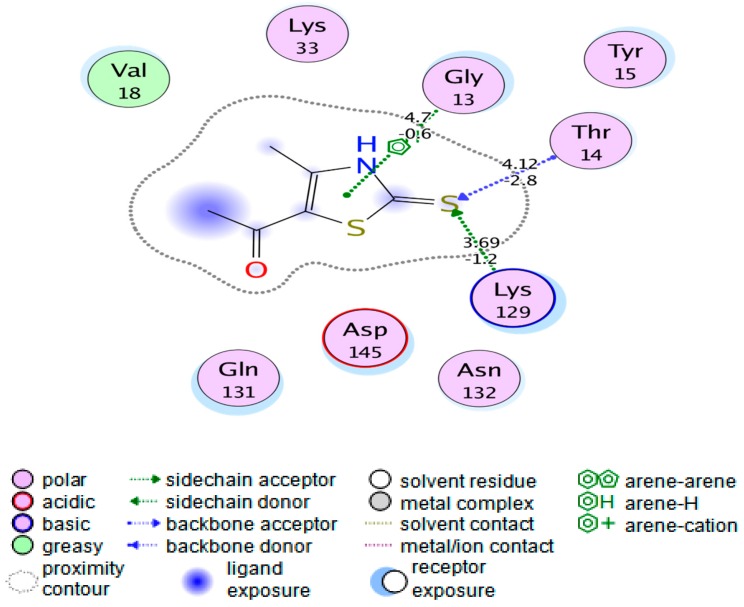
Thiazoline **2c** into the binding pocket of CDK 2 enzyme showing the binding energies and interactions with amino acid residues.

The binding affinity of thiazoline **2c** showed hydrogen acceptor interactions with **Thr 14** and **Lys 129**, with binding energies equal −4 (kcal/mol). Also, it exhibited a pi-hydrogen interaction with **Gly 13**, with binding energy equal −0.6 (kcal/mol) (Figure 3).

**Figure 4:**
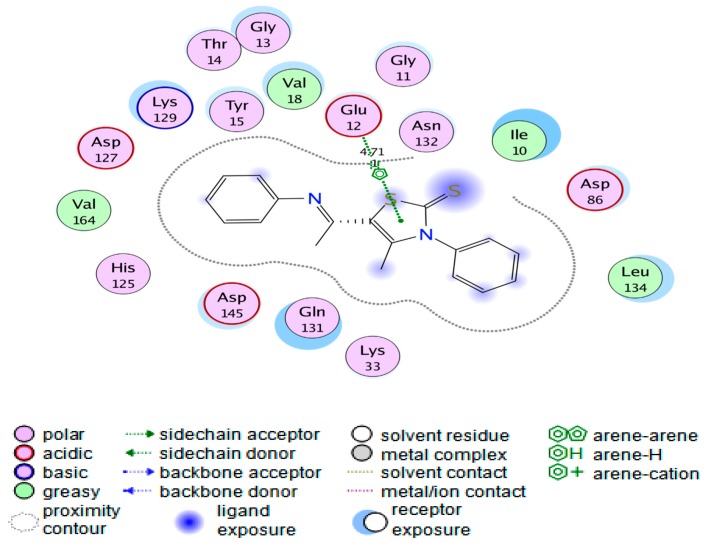
Thiazoline **5b** into the binding pocket of CDK 2 enzyme showing the binding energies and interactions with amino acid residues.

In contrast, thiazoline derivative **5b** had less binding affinity to the CDK 2 enzyme than compound **2c**. It showed only one pi-hydrogen interaction with **Glu 12**, with binding energy equal -0.1 (kcal/mol) (Figure 4).

#### 2.3.3. Antitumor Evaluation of Some Selected Examples of the Synthesized Compounds

**Figure 5:**
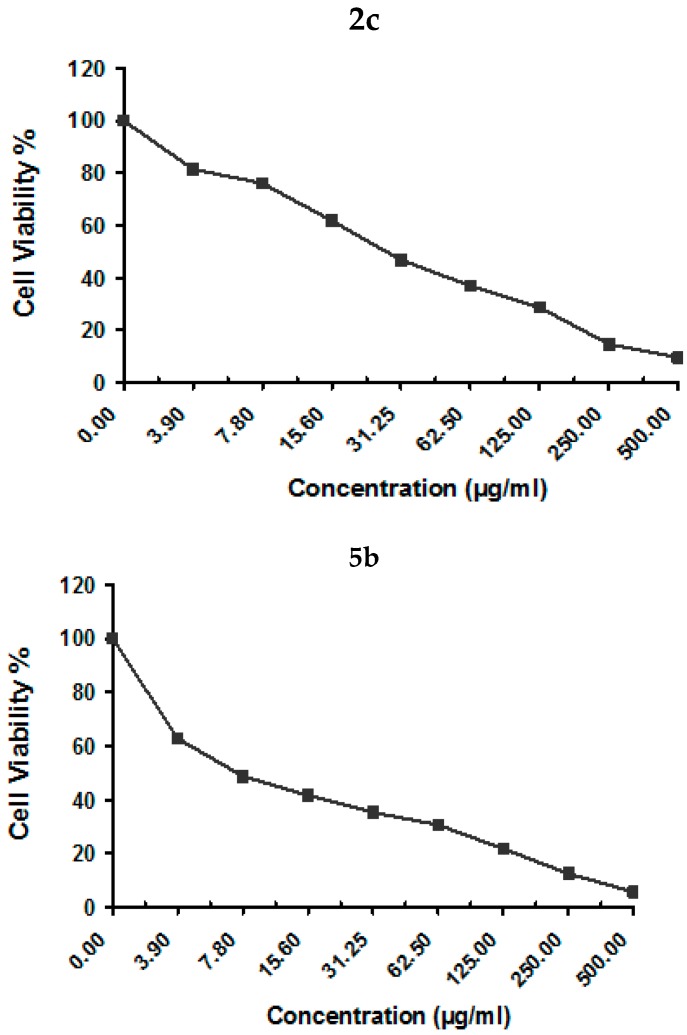
The viability values of the most active thiazolines against HepG-2 cell line.

**Figure 6:**
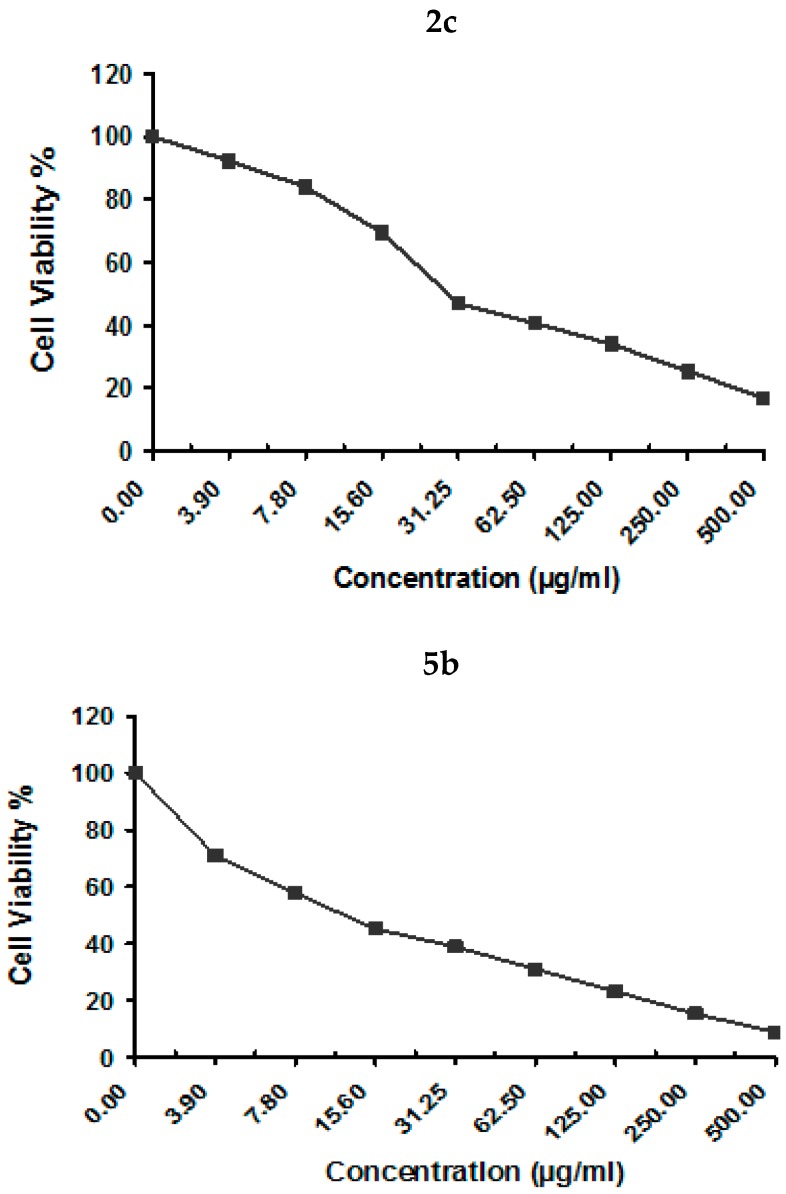
The viability values of the most active thiazoline derivatives against HCT-116 cell line.

The in vitro antitumor activity of some selected examples of the synthesized thiazolines and the reference drug Doxorubicin were investigated using the MTT method [55] against human hepatocellular carcinoma cell line (HepG-2) and colon carcinoma cells (HCT-116). The concentration of the tested thiazolines needed to inhibit 50% of the cells population (IC_50_) was calculated and is presented in Table 5 and Table 6 and Figure 5 and Figure 6. 

The results of Table 5 and Table 6 showed that the tested thiazolines **5b** and **2c** have the highest effectiveness compared to the other thiazoline derivatives against the HepG-2 and HCT-116 cell lines, with IC_50_ values of approximately 7.46 µg/mL and 27.9 µg/mL for HepG-2 and 12.6 µg/mL and 29.1 µg/mL, for HCT-116, respectively. The remaining compounds have a noticeably moderated efficiency.

## 3. Materials and Methods 

### 3.1. Chemistry 

#### 3.1.1. General Information

All the melting points were measured using a Gallenkamp apparatus (Thermo Fisher Scientific, Paisley, UK) in open glass capillaries and are uncorrected. Infrared spectra (IR) were recorded using the KBr disc technique on a Perkin Elmer FT-IR spectrophotometer 1000 (PerkinElmer, Waltham, MA, USA). NMR spectra (^1^H and ^13^C) were measured using an ECP 400 NMR spectrometer (JEOL, Tokyo, Japan) operating at 400 MHz in deuterated chloroform (CDCl_3_). Mass spectra were measured on a Shimadzu GCMS-QP 1000 EX mass spectrometer (Tokyo, Japan) at 70 eV. Elemental analysis were recorded on a 2400 CHN Elemental Analyzer. The single-crystal X-ray diffraction measurements were done on a SMART APEX II CCD diffractometer (Bruker AXS Advanced X-ray Solutions GmbH, Karlsruhe, Germany). The biological assessments of the synthesized compounds were done in the Medical Mycology Laboratory of the Regional Center for Mycology and Biotechnology of Al-Azhar University, Cairo, Egypt. The 2-oxo-*N*′-phenylpropanehydrazonoyl chloride (**6**) was prepared as described in the literature [49,50].

#### 3.1.2. The Synthetic Procedure for the Target Thiazolines **2a**–**e**

To a solution of 3-chloropentane-2,4-dione (0.134 g, 0.112 mL, 1 mmol) or ethyl 2-chloro-3-oxobutanoate (0.164 g, 0.138 mL, 1 mmol) in ethanol (15 mL), carbon disulfide (0.152 g, 0.12 mL, 2 mmol) and the appropriate amine derivative (1 mmol) were added. The reaction mixture was stirred for six hours. The solid product that formed was filtered and washed with ethanol, and recrystallized to afford the corresponding thiazolines **2a**–**e**. The physical properties and spectroscopic data of compounds **2a**–**d** are in agreement with the literature [45,47,48].

*1-(4-Methyl-3-phenyl-2-thioxo-2,3-dihydrothiazol-5-yl)ethan-1-one* (**2a**): Yellow solid, (0.23 g, yield 92%); m.p. 172–174 °C (EtOH) [lit mp. 171–174 °C [45,47]]; IR v_max_ 1630 (C=O), 1490 (C=S) cm^−1^; ^1^H-NMR (CDCl_3_) δ 2.25 (s, 3H, CH_3_), 2.35 (s, 3H, CH_3_), 7.14–7.52 (m, 5H, Ph); ^13^C-NMR (CDCl_3_) δ 16.14, 30.23 (2CH_3_), 112.50, 147.34, 121.50, 128.05, 130.16, 137.08, 188.17, 190.00. Anal. Calcd. for C_12_H_11_NOS_2_ (249.35): C, 57.80; H, 4.45; N, 5.62. Found: C, 57.91; H, 4.52; N, 5.55%.

*Ethyl 4-methyl-3-phenyl-2-thioxo-2,3-dihydrothiazole-5-carboxylate* (**2b**): white powder, (0.24 g, yield 85%); m.p. 160–162 °C (EtOH) [lit. mp. 158–160 °C [47]]; IR v_max_ 1698 (C=O), 1621 (C=C), 1514 (C=S) cm^−1^; ^1^H-NMR (CDCl_3_) δ 1.29 (t, 3H, CH_3_), 2.25 (s, 3H, CH_3_), 4.25 (q, 2H, CH_2_), 7.15–7.53 (m, 5H, Ph); MS (*m/z*) (%) 279 (M^+^, 100%), 278 (54%), 250 (59%), 234 (12%). Anal. Calcd. for C_13_H_13_NO_2_S_2_ (279.38): C, 55.89; H, 4.69; N, 5.01. Found: C, 55.82; H, 4.73; N, 5.10%.

*1-(4-Methyl-2-thioxo-2,3-dihydrothiazol-5-yl)ethanone* (**2c**): white powder, (0.147 g, yield 85%); m.p. 208–210 °C (EtOH) [lit. mp. 210–211 °C [48]]; IR v_max_ 3210 (NH), 1669 (C=O), 1617(C=C), 1537(C=S) cm^−1^; ^1^H-NMR (CDCl_3_) δ 1.77 (s, 3H, CH_3_), 2.35 (s, 3H, CH_3_), 7.2 (s, H, NH); ^13^C-NMR (CDCl_3_) δ 17.70 (CH_3_), 25.60 (CH_3_), 119.00, 148.00, 188.00, 192.00; MS (*m/z*) (%) 173 (M^+^, 2%), 43 (34%). Anal. Calcd. for C_6_H_7_NOS_2_ (173.26): C, 41.59; H, 4.07; N, 8.08. Found: C, 41.63; H, 4.14; N, 8.12%.

*Ethyl 4-methyl-2-thioxo-2,3-dihydrothiazole-5-carboxylate* (**2d**): white powder, (0.152 g, yield 75%); m.p. 146–148 °C (EtOH) [lit. mp. 151–152 °C [48]]; IR v_max_ 3383 (NH), 1674 (C=O), 1601 (C=C), 1425 (C=S) cm^−1^; ^1^H-NMR (CDCl_3_) δ 1.36 (t, 3H, CH_3_), 2.49 (s, 3H, CH_3_), 4.35 (q, 2H, CH_2_), 7.19 (s, H, NH); ^13^C-NMR (CDCl_3_) δ 14.22 (CH_3_), 29.34 (CH_3_), 61.94 (CH_2_), 118.50, 162.81, 177.50, 193.18; MS (*m/z*) (%) 204 (16%), 203 (M^+^, 70%), 159 (99%), 158 (30%), 43 (100%). Anal. Calcd. for C_7_H_9_NO_2_S_2_ (203.28): C, 41.36; H, 4.46; N, 6.89. Found: C, 41.42; H, 4.53; N, 6.78%.

*Ethyl 4-methyl-3-(phenylamino)-2-thioxo-2,3-dihydrothiazole-5-carboxylate* (**2e**): white powder, (0.25 g, yield 85%); mp 210–212 °C (EtOH); IR v_max_ 3390 (NH), 1701 (C=O), 1593 (C=C), 1544 (C=S) cm^−1^; ^1^H-NMR (CDCl_3_) δ 1.33 (t, 3H, CH_3_), 2.45 (s, 3H, CH_3_), 4.30 (q, 2H, CH_2_), 7.21–7.69 (m, 5H, ArH), 11.85 (s, 1H, NH); MS (*m/z*) (%) 294 (M^+^, 1%), 248 (6%), 216 (80%), 77 (100). Anal. Calcd. for C_13_H_14_N_2_O_2_S_2_ (294.39): C, 53.04; H, 4.79; N, 9.52. Found: C, 53.12; H, 4.84; N, 9.43%. 

#### 3.1.3. Synthetic Procedure for Substituted 4-Methyl-3-Phenylthiazole-2(3*H*)-Thione **5a**–**e**

A mixture of the appropriate amine (1 mmol) and thiazoline **2a** (0.249 g, 1 mmol) in ethanol (10 mL) was refluxed for approximately six hours until the precipitation was produced. Then, the product was filtered and recrystallized from ethanol to afford the corresponding condensation product. 

*5-(1-Hydrazonoethyl)-4-methyl-3-phenylthiazole-2(3H)-thione* (**5a**): Yellow powder, (0.145 g, yield 55%); m.p. 215–217 °C (EtOH); IR v_max_ 3208, 3160 (NH_2_), 1631 (C=N), 1487 (C=S) cm^−1^; ^1^H-NMR (CDCl_3_) δ 1.99 (s, 3H, CH_3_), 2.49 (s, 3H, CH_3_), 6.65 (s, 2H, NH_2_), 7.30–7.60 (m, 5H, Ph); MS (*m/z*) (%) 264 (22%), 263 (M^+^, 82%),77(100%). Anal. Calcd. for C_12_H_13_N_3_S_2_ (263.38): C, 54.72; H, 4.97; N, 15.95. Found: C, 54.68; H, 4.87; N, 15.88%. 

*4-Methyl-3-phenyl-5-(1-(phenylimino)ethyl)thiazole-2(3H)-thione* (**5b**): Yellow powder, (0.162 g, yield 50%; m.p. 228–230 °C (EtOH); IR v_max_ 1590 (C=N), 1492 (C=S) cm^−1^; ^1^H-NMR (CDCl_3_) δ 1.95 (s, 3H, CH_3_), 2.17 (s, 3H, CH_3_), 7.30-7.59 (m, 10H, Ph); MS (*m/z*) (%) 324 (M^+^, 2%), 247 (24%), 77(100%). Anal. Calcd. for C_18_H_16_N_2_S_2_ (324.46): C, 66.63; H, 4.97; N, 8.63. Found: C, 66.55; H, 4.83; N, 8.55%. 

*(E)-4-Methyl-3-phenyl-5-(1-(2-phenylhydrazono)ethyl)thiazole-2(3H)-thione* (**5c**): Yellow powder, (0.203 g, yield 60%); m.p. 220–222 °C (EtOH); IR v_max_ 3289 (NH), 1589 (C=N), 1495 (C=S) cm^−1^; ^1^H-NMR (CDCl_3_) δ 2.08 (s, 3H, CH_3_), 3.32 (s, 3H, CH_3_), 7.12–7.58 (m, 10H, ArH), 9.51 (s, 1H, NH); ^13^C-NMR (CDCl_3_) δ 15.38 (CH_3_), 15.71 (CH_3_), 112.71, 137.96, 119.31, 124.02, 123.81, 128.57, 128.96, 134.30, 135.47, 137.96, 145.27, 186.91; MS (*m/z*) (%) 340 (20%), 339 (M^+^, 96%), 247 (14%), 77(100%). Anal. Calcd. for C_18_H_17_N_3_S_2_ (339.48): C, 63.68; H, 5.05; N, 12.38. Found: C, 63.73; H, 5.12; N, 12.29%. 

*5-(1-((1H-1,2,4-Triazol-5-yl)imino)ethyl)-4-methyl-3-phenylthiazole-2(3H)-thione* (**5d**): Yellow powder, (0.158 g, yield 50%); m.p. 275–277 °C (DMF); IR v_max_ 3370 (NH), 1637 (C=N), 1577(C=C), 1470 (C=S) cm^−1^; ^1^H-NMR (CDCl_3_) δ 1.95 (s, 3H, CH_3_), 2.20 (s, 3H, CH_3_), 7.30-7.62 (m, 6H, Ph), 8.72 (s, 1H, NH); MS (*m/z*) (%) 315 (M^+^, 6%), 300 (10%), 105 (68%). Anal. Calcd. for C_14_H_13_N_5_S_2_ (315.42): C, 53.31; H, 4.15; N, 22.20. Found: C, 53.28; H, 4.22; N, 22.12%. 

*5-(1-((1H-Benzo[d]imidazol-2-yl)imino)ethyl)-4-methyl-3-phenylthiazole-2(3H)-thione* (**5e**): Yellow powder, (0.164 g, yield 45%); m.p. 200–202 °C (EtOH); IR v_max_ 3236 (NH), 1586 (C=C), 1487 (C=S) cm^−1^; ^1^H-NMR (CDCl_3_) δ 1.90 (s, 3H, CH_3_), 2.25 (s, 3H, CH_3_), 7.20–7.61 (m, 9H, Ph), 11.62 (s, 1H, NH); MS (*m/z*) (%) 364 (M^+^, 8%), 249 (52%), 248 (36%), 133 (98%), 43 (100%). Anal. Calcd. for C_19_H_16_N_4_S_2_ (364.49): C, 62.61; H, 4.42; N, 15.37. Found: C, 62.55; H, 4.38; N, 15.42%. 

#### 3.1.4. 3,7-Diacetyl-8-methyl-1,9-diphenyl-4,6-dithia-1,2,9-triazaspiro[4.4]nona-2,7-diene (**7**)

To a mixture of thiazole derivative **2a** (0.249g, 1 mmol) and 2-oxo-*N*′-phenylpropane hydrazonoyl chloride (**6**) (0.196 g, 1 mmol) in dry benzene (10 mL), triethylamine (0.2 g, 0.28 mL, 2 mmol) was added and the reaction mixture was heated under reflux for 6 h. The precipitated triethylamine hydrochloride was removed by filtration and the filterate was evaporated under a vacuum. The remaining residue was treated using ethanol and the solid product that formed was filtered off and recrystallized using ethanol to give the spiro-compound **7**. Yellowish powder, (0.204 g, yield 50%); m.p. 154–156 °C (DMF)[Lit mp. 155–157 °C (MeCN)] [51]]; IR v_max_ 1644 (C=O), 1549 (C=C) cm^−1^; ^1^H-NMR (CDCl_3_) δ 1.92 (s, 3H, CH_3_), 2.10 (s, 3H, CH_3_), 2.22 (s, 3H, CH_3_), 7.35–7.59 (m, 10H, Ph); MS (*m/z*) (%) 409 (M^+^, 2%), 43 (100%). Anal. Calcd. for C_21_H_19_N_3_O_2_S_2_ (409.52): C, 61.59; H, 4.68; N, 10.26. Found: C, 61.66; H, 4.73; N, 10.22%.

### 3.2. X-ray Analysis 

The thiazoline derivative **5c** was obtained as single crystals by slow evaporation from an ethanol solution of the pure compound at room temperature. The thiazoline structure was evaluated using SHELXT [56,57]. All the crystallographic data of the crystal structure **5c** are available and can be obtained free of charge from the Cambridge Crystallographic Data Centre via www.ccdc.cam.ac.uk/data_request/cif. For additional details, refer to the Supplementary Materials file.

### 3.3. Biological Evaluations

#### 3.3.1. The In Vitro Antimicrobial Investigation

The antimicrobial activities of the newly synthesized thiazolines were evaluated by the inhibition zone technique on *Aspergillus fumigatus*, *Candida albicans*, *Staphylococcus aureus*, *Bacillus subtilis*, *Salmonella* sp. and *Escherichia coli* [53,54]. Additional details are available in the Supplementary Materials file.

#### 3.3.2. In Vitro Cytotoxic Activity

The cytotoxic assessment of the target thiazole derivatives was carried out against two cancer cell lines (HepG2 and HCT-116) using the MTT assay after 24 h of incubation [55]. The experimental procedure is included in the Supplementary Materials file.

### 3.4. Molecular Modeling

The docking study was performed using the MOE 2014.09 software [58]. Regularization and optimization for the protein and ligand were performed. Each docked thiazole was assigned a score according to its fit in the ligand binding pocket (LBP) and its binding mode.

## 4. Conclusions

In this work, new thiazolines were prepared, characterized and evaluated for their biological activities. The results of the antimicrobial evaluation indicated that the thiazoline derivatives **2c**, **5b** and **5e** exhibited high inhibitory activity against *Salmonella* sp., while compounds **2e** and **5b** were comparable to Gentamycin against *Escherichia coli*. The data from the in vitro antitumor evaluation revealed that the thiazolines **5b** and **2c** were the most effective against the HepG-2 and HCT-116 cell lines. This remarkable efficacy has potential usage in numerous pharmaceutical applications. Molecular docking study supported anticancer results and showed binding affinities for thiazolines **5b** and **2c** towards cyclin-dependant kinase 2.

## Figures and Tables

**Table 1:** The crystal and experimental data of thiazoline **5c**.

**Crystal Data**	
Chemical formula	C_18_H_17_N_3_S_2_
Mr	339.46
Crystal system, space group	Monoclinic, *Pc*
Temperature (K)	293
*a*, *b*, *c* (Å)	9.2105 (4), 7.4969 (3), 12.9869 (5)
β (°)	109.440 (2)
V (Å^3^)	845.62 (6)
*Z*	2
Radiation type	Cu *K*α
µ (mm^−1^)	2.86
Crystal size (mm)	0.42 × 0.35 × 0.32
**Data Collection**	
Diffractometer	Bruker APEX-II D8 venture diffractometer
Absorption correction	Multi-scan SADABS Bruker 2014
T_min_, T_max_	0.884, 0.909
No. of measured, independent and observed [I > 2σ(I)] reflections	8683, 3089, 2993
R_int_	0.039
**Refinement**	
R[*F^2^* > 2σ( *F^2^*)], wR( *F^2^*), S	0.030, 0.075, 1.07
No. of reflections	3089
No. of parameters	214
No. of restraints	2
H-atom treatment	H atoms treated by a mixture of independent and constrained refinement
Δρ_max_, Δρ_min_ (e Å^−3^)	0.20, −0.32
Absolute structure	Flack x determined using 1376 quotients [(I+) − (I)]/ [(I+) + (I−)]
Flack parameter	0.031 (9)

**Table 2:** Selected geometric parameters (Å) of thiazoline **5c**.

S1—C1	1.722 (3)	N1—C4	1.447 (4)
S1—C2	1.748 (3)	N2—N3	1.363 (4)
S2—C1	1.668 (3)	N2—C11	1.289 (4)
N1—C1	1.363 (4)	N3—C13	1.390 (4)
N1—C3	1.413 (4)		
C1—S1—C2	92.77 (14)	S1—C2—C11	116.7 (2)
C1—N1—C3	115.5 (2)	N1—C3—C2	112.2 (3)
C1—N1—C4	121.2 (3)	N1—C3—C10	118.1 (3)
C3—N1—C4	123.3 (2)	N1—C4—C5	118.0 (3)
N3—N2—C11	119.6 (3)	N1—C4—C9	120.2 (2)
N2—N3—C13	118.4 (3)	N2—C11—C2	112.3 (3)
S1—C1—N1	109.1 (2)	N2—C11—C12	125.7 (3)
S2—C1—N1	127.2 (2)	N3—C13—C14	118.1 (3)
S1—C1—S2	123.69 (17)	N3—C13—C18	122.5 (3)
S1—C2—C3	110.4 (2)		

**Table 3:** Hydrogen-bond geometry (Å) of thiazoline **5c**.

D—H···A	D···A	D—H···A
N3—H1···S2^i^	3.579 (3)	144 (3)
Symmetry codes: (i) x − 1, −y + 1, z − 1/2.		

**Table 4:** The in vitro antimicrobial assessment of the synthesized compounds expressed as inhibition zones diameter in millimeters (mm).

Sample	Microorganisms					
	Fungi		Gram Positive Bacteria		Gram Negative Bacteria	
	*AF*	*CA*	*SA*	*BS*	*SSP*	*EC*
**2b**	NA	NA	NA	NA	11	8
**2c**	14	13	15	12	15	14
**2d**	10	13	13	10	13	14
**2e**	15	15	13	14	13	17
**5a**	NA	13	9	9	13	12
**5b**	NA	13	9	10	14	16
**5c**	NA	12	9	9	12	10
**5d**	13	17	13	12	14	13
**5e**	NA	14	8	9	13	9
**Amphotericin B**	23	25	-	-	-	-
**Ampicillin**			23	32	-	-
**Gentamycin**	-	-	-	-	17	19

NA: No activity; results of the antimicrobial evaluation are expressed as mean of inhibition zone diameter (mm) for different compounds tested in triplicate; *Aspergillus fumigatus (RCMB 002008 (4)* (AF), *Candida albicans (RCMB 05036) (CA*), *Staphylococcus aureus (RCMB010010) (SA*), *Bacillus subtilis (RCMB 010067)* (*BS*), *Salmonella sp. (RCMB 010043) (SSP*), *Escherichia coli* *(RCMB 010052* *(EC)*.

**Table 5:** Viability values and IC_50_ of tested thiazolines and control drug *Doxorubicin* against *hepatocellular carcinoma cell line (HepG-2)*.

Sample Number	Sample Concentration (μg/mL) Viability %									IC_50_ (µg/mL)
	500	250	125	62.5	31.25	15.6	7.8	3.9	0	
**Doxorubicin (standard)**	2.08	3.36	4.86	6.51	11.04	19.38	24.82	28.86	100	0.36
**2c**	9.56	14.65	28.72	36.93	46.80	61.78	76.09	81.43	100	27.9
**2d**	14.96	25.37	35.18	46.85	62.34	76.82	84.17	90.64	100	56.1
**5a**	17.39	35.26	46.87	62.34	79.15	87.29	94.12	97.34	100	112
**5b**	5.72	12.46	21.79	30.69	35.28	41.63	48.76	62.81	100	7.46
**5d**	10.67	24.16	36.25	48.32	61.74	76.98	85.04	92.36	100	58.6

**Table 6:** Viability values and IC_50_ of assessed thiazolines and *Doxorubicin* against *colon carcinoma cells (HCT-116)* Cell Line.

Sample Number	Sample Concentration (μg/mL) Viability %									IC_50_ (µg/mL)
	500	250	125	62.5	31.25	15.6	7.8	3.9	0	
**Doxorubicin (standard)**	2.08	3.36	4.86	6.51	11.04	19.38	24.82	28.86	100	0.49
**2c**	16.72	25.46	33.95	40.67	46.98	69.41	84.02	92.37	100	29.1
**2d**	21.53	32.68	40.97	56.13	71.84	86.25	93.89	97.04	100	87.8
**5b**	8.94	15.36	23.21	31.05	38.92	45.18	57.85	71.06	100	12.6
**5d**	17.85	32.64	45.72	57.18	72.34	87.29	94.12	97.65	100	102

## Supplementary Materials

Online supplementary information includes detailed methods of the X-ray analysis, along with cytotoxic, and antimicrobial evaluations.

## References

[1] Marson C.M., Matthews C.J., Yiannaki E., Atkinson S.J., Soden P.E., Shukla L., Lamadema N., Thomas N.S. (2013). Discovery of potent, isoform-selective inhibitors of histone deacetylase containing chiral heterocyclic capping groups and a *N*-(2-aminophenyl)benzamide binding unit. J. Med. Chem..

[2] Patil P.A., Amnerkar N.D., Pathare S.S., Bhusari K.P. (2015). Docking study of p-hydroxybenzohydrazide derivatives as tyrosine kinase inhibitors and anticancer agents. J. Comput. Methods Mol. Des..

[3] Wang W., Zhao B., Xu C., Wu W. (2012). Synthesis and antitumor activity of the thiazoline and thiazine multithioether. Int. J. Org. Chem..

[4] Sondhi S.M., Johar M., Singh N., Dastidar S.G. (2004). Synthesis of biscoupled hemin-thiazoline derivatives and their anticancer activity evaluation. Indian J. Chem..

[5] Mahani N.M., Sabermahani F., Jahani P.M., Jalali N. (2015). A density function theory based quantitative structure activity relationships study of thiazoline derivatives as anticancer agents. Iran. J. Anal. Chem..

[6] Taher A.T., Khalil N.A., Ahmed E.M. (2011). Synthesis of novel isatin-thiazoline and isatin-benzimidazole conjugates as anti-breast cancer agents. Arch. Pharm. Res..

[7] Miller D.D., Dalton J.T., Gududuru V., Hurh E. (2005). Thiazoline analogs as cell proliferation inhibitors. Chem. Abstr..

[8] Ng R.A., Sui Z. (2005). Preparation of thiazoline derivatives as selective androgen receptor modulators (SARMs). Chem. Abstr..

[9] Pine M.J., Mirand E.A., Ambrus J.L., Bock F.G. (1983). Antitumor studies of 2- amino-2-thiazoline and other tumor-modifying agents. J. Med..

[10] Randazzo A., Bifulco G., Giannini C., Bucci M., Debitus C., Cirino G., Gomez-Paloma L. (2001). Halipeptins A and B: Two novel potent anti-inflammatory cyclic depsipeptides from the Vanuatu marine sponge Haliclona species. J. Am. Chem. Soc..

[11] Sondhi S.M., Rani R., Gupta P.P., Agrawal S.K., Saxena A.K. (2009). Synthesis, anticancer, and anti-inflammatory activity evaluation of methanesulfonamide and amidine derivatives of 3,4-diaryl-2-imino-4-thiazolines. Mol. Divers..

[12] Omar A.M., Ahmed I.C., Hassan A.M., AboulWafa O.M., Abou-Shleib H., Ismail K.A. (1990). Synthesis and evaluation for antibacterial and antifungal activities of new 1-phenylhydrazono-2-(substituted thiocarbamoyl)hydrazonopyruvaldehyde and the corresponding thiazoline and thiazolidinone derivatives. Alex. J. Pharm. Sci..

[13] Shih M., Ke F. (2004). Synthesis and evaluation of antioxidant activity of sydnonyl substituted thiazolidinone and thiazoline derivatives. Bioorg. Med. Chem..

[14] Makhaeva G.F., Boltneva N.P., Lushchekina S.V., Serebryakova O.G., Stupina T.S., Terentiev A.A., Serkov I.V., Proshin A.N., Bachurin S.O., Richardson R.J. (2016). Synthesis, molecular docking and biological evaluation of *N*,*N*-disubstituted 2-aminothiazolines as a new class of butyrylcholinesterase and carboxylesterase inhibitors. Bioorg. Med. Chem..

[15] Lesyk R.B., Zimenkovsky B.S. (2004). 4-Thiazolidones: Centenarian history, current status and perspectives for modern organic and medicinal chemistry. Curr. Org. Chem..

[16] Malik S., Upadhyaya P.K., Miglani S. (2011). Thiazolidinediones: A plethro of biological load. Int. J. Pharm. Tech. Res..

[17] Fakhari A.R., Hosseiny Davarani S.S., Ahmar H., Makarem S. (2008). Electrochemical study of catechols in the presence of 2-thiazoline-2-thiol: Application to electrochemical synthesis of new 4,5-dihydro-1,3-thiazol-2-ylsulfanyl-1,2-benzenediol derivatives. J. Appl. Electrochem..

[18] Nematollahi D., Tammari E. (2005). Electroorganic Synthesis of catecholthioethers. J. Org. Chem..

[19] Laurence C., El Ghmari M.J., Le Questel J.Y., Berthelot M., Mokhlisse R. (1998). Structure and molecular interactions of anti-thyroid drugs. Part 3.1 Methimazole: A diiodine sponge. J. Chem. Soc. Perkin Trans. 2.

[20] Thomes J.C., Comby F., Lagorce J.F., Buxeraud J., Raby C. (1992). Sites of action of 2-thiazoline-2-thiol on biogenesis of thyroid hormones. Jpn. J. Pharmacol..

[21] Almasirad A., Nassiri Koopaei M., Shafiee A., Nassiri N., Javad Assarzadeh M., Tabei A., Ghadim M. (2012). Synthesis of new 1,3-thiazoline-2-thiones as potential antimycobacterial agents. J. Pharm. Health Sci..

[22] Teng Y., Wang X., Zou L., Huang M., Du X. (2015). Experimental and theoretical study on the binding of 2-mercaptothiazoline to bovine serum albumin. J. Lumin..

[23] Zou L., Zhang X., Shao M., Sun R., Zhu Y., Zou B., Huang Z., Liu H., Teng Y. (2019). A biophysical probe on the binding of 2-mercaptothioazoline to bovine hemoglobin. Environ. Sci. Pollut. Res. Int..

[24] Gawron O., Keil L. (1960). Competitive inhibition of acetylcholinesterase by several thiazolines and oxazolines. Arch. Biochem. Biophys..

[25] Handrick G.R., Atkinson E.R., Granchelli F.E., Bruni R.J. (1965). Potential antiradiation drugs. II. 2-Amino-1-alkanethiols, 1-amino-2-alkanethiols, 2-thiazolines, and 2-thiazoline-2-thiols. J. Med. Chem..

[26] Mahal H.S., Mukherjee T. (1999). Kinetic and spectroscopic properties of intermediates formed by the reaction of some oxidizing and reducing radicals with 2-mercaptothiazoline (2-MT) in aqueous solutions. Radiat. Phys. Chem..

[27] Fang C.L. (1996). General Concepts of Additives in the Electroplating Solution.

[28] Little L.H., Ottewill R.H. (1962). Studies on the infrared spectra of a mercaptotriazole and mercaptothiazoline and their adsorption on silver iodide. Can. J. Chem..

[29] Solmaz R., Kardas G., Culha M., Yazici B., Erbil M. (2008). Investigation of adsorption and inhibitive effect of 2-mercaptothiazoline on corrosion of mild steel in hydrochloric acid media. Electrochim. Acta.

[30] Filho N.L.D., do Carmo D.R., Gessner F., Rosa A.H. (2005). Preparation of a clay-modified carbon paste electrode based on 2-thiazoline-2-thiol-hexadecylammonium sorption for sensitive determination of mercury. Anal. Sci..

[31] Eun J.S., Kim K.S., Kim H.N., Park S.A., Ma T.-Z., Lee K.A., Kim D.K., Kim H.K., Kim I.S., Jung Y.H. (2007). Synthesis of psoralen derivatives and their blocking effect of hKv1.5 channel. Arch. Pharm. Res..

[32] Mori M., Takagi M., Noritake C., Kagabu S. (2008). 2,4-Dioxo-1, 3-thiazolidine derivatives as a lead for new fungicides. J. Pestic. Sci..

[33] Sahu S.K., Banerjee M., Mishra S.K., Mohanta R.K., Panda P.K., Misro P.K. (2007). Synthesis, partition coefficients and antibacterial activity of 3′-phenyl(substituted)-6′-aryl-2′(1*H*)cis-3′,3′a-dihydrospiro[3-H-indole-3,5′-pyrazolo (3′,4′-d)thiazolo-2-(1*H*)-ones]. Acta Pol. Pharm..

[34] Dwivedi C., Gupta T.K., Parmar S.S. (1972). Substituted thiazolidones as anticonvulsants. J. Med. Chem..

[35] Verma A., Saraf S.K. (2008). 4-Thiazolidinone—A biologically active scaffold. Eur. J. Med. Chem..

[36] Nabih I., El-Hawary F., Zoorob H. (1972). Structure and activity of thiazole-type schistosomicidal agents. J. Pharm. Sci..

[37] Nelson P.A., Paulson G.D., Feil V.J. (1987). The effect of nitrite on ^14^C-sulphathiazole (4-amino-*N*-2-thiazolyl[U-^14^C]benzenesulphonamide) metabolism in the rat. Xenobiotica.

[38] Toplak R.B., Rocherulle P., Lorcy D. (2002). Unexpected reaction of dimethoxycarbonyl dithiole-2-thione or tetramethoxycarbonyl TTF as dipolarophiles. Tetrahedron Lett..

[39] Humphlett W.J., Lamon R.W. (1964). 4-Thiazoline-2-thiones. I. The Structure of intermediate 4-hydoxythiazolidine-2-thiones. J. Org. Chem..

[40] Humphlett W.J., Lamon R.W. (1964). 4-Thiazoline-2-thiones. II. Preparation of 4-alkylsulfonylmethyl derivatives. J. Org. Chem..

[41] Lamon R.W., Humphlett W.J. (1967). 4-Thiazoline-2-thiones. IV. Preparation from amino acids. J. Heterocycl. Chem..

[42] Dunn A.D., Rudorf W. (1989). Carbon Disulphide in Organic Chemistry.

[43] Ze-Mei G., Qin L., Shao-Jun Z., Tie-Ming C., Yu-Xin C., Run-Tao L. (2006). Facile One-pot Synthesis of Ethyl 3-Alkyl-4-hydroxy-2-thioxothiazolidine-4-carboxylates. Chin. J. Chem..

[44] Arab-Salmanabadi S. (2017). Synthesis and spectral characterization of novel bis-thiazole derivatives via ring closure of benzo[d]thiazol-2-amine, various α-haloketones, and S-nucleophiles. J. Heterocycl. Chem..

[45] Yavari I., Sirouspour M., Souri S. (2010). A one-pot synthesis of *N*-alkylthiazoline-2-thiones from CS_2_, primary amines, and 2-chloro-1,3-dicarbonyl compounds in water. Monatsh. Chem..

[46] Iravani N., Karami B., Asadimoghaddam F., Monfared M. (2012). Solvent free synthesis of 1-[3-alkyl-4-methy-2-thioxo-2,3-dihydrothiazole-5-yl]-ethanone in a multicomponent reaction. J. Sulfur Chem..

[47] Janikowska K., Makowiec S. (2011). Simple Method for the Preparation of Dialkyl (2,3-Dihydro-1,3-thiazol-2-YL)-phosphonates. Phosphorus Sulfur Silicon Relat. Elem..

[48] D’xmico J.J. (1953). Thiazolethiols and their Derivatives. J. Am. Chem. Soc..

[49] Dieckmann W., Platz L. (1905). Ueber eine neue bildungsweise von osotetrazonen. Ber. Dtsch. Chem. Ges..

[50] Abushamleh A.S., Al-Aqarbeh M.M., Day V. (2008). Transition metal complexes of derivatized chiral dihydro-1,2,4-triazin-6-ones. Template synthesis of nickel (II) tetraaza-(4*N*-M) complexes incorporating the triazinone moiety. Am. J. Appl. Sci..

[51] Budarina E.V., Dolgushina T.S., Petrov M.I., Labelish N.N., Kol’tsov A.A., Bel’skii V.K. (2007). Heterocyclic thiones and their analogs in 1,3-dipolar cycloaddition: VII, reactions of 4-methy-1,3-thiazole-2(3*H*)-thiones with nitrile imines. Russ. J. Org. Chem..

[52] Allen F.H., Kennard O., Watson D.G., Brammer L., Orpen A.G., Taylor R. (1987). Tables of bond lengths determined by X-ray and neutron diffraction. Part 1. Bond lengths in organic compounds. J. Chem. Soc. Perkins Trans..

[53] Clinical and Laboratory Standards Institute (2012). Performance Standards for Antimicrobial Susceptibility Testing.

[54] Ghorab M.M., Alsaid M.S., El-Gaby M.S.A., Safwat N.A., Elaasser M.M., Soliman A.M. (2016). Biological evaluation of some new *N*-(2,6-dimethoxypyrimidinyl)thioureido benzenesulfonamide derivatives as potential antimicrobial and anticancer agents. Eur. J. Med. Chem..

[55] Mosmann T. (1983). Rapid colorimetric assay for cellular growth and survival: Application to proliferation and cytotoxicity assays. J. Immunol. Methods.

[56] Sheldrick G.M. (2008). A short history of SHELX. Acta Crystallogr..

[57] Sheldrick G.M. (2015). SHELXT–Integrated space-group and crystal-structure determination. Acta Crystallogr. Sect. A Found. Adv..

[58] (2015). Molecular Operating Environment (MOE), 2014.09.

