# Supplementary material for: Synthesis, X-ray Analysis, Biological Evaluation and Molecular Docking Study of New Thiazoline Derivatives
Source: Molecules. 2019 Apr 26;24(9):1654. doi: 10.3390/molecules24091654 (PMC6540005; doi:10.3390/molecules24091654)
Supplement: Supplementary file 1 [file molecules-24-01654-s001.pdf]

Supplementary File

Synthesis, X-Ray Analysis, Biological Evaluation and Molecular Docking Study of New Thiazoline Derivatives

Yahia N. Mabkhot [1,*], H. Algarni [2,3], Abdulrhman Alsayari [4], Abdullatif Bin Muhsinah [4], Nabila A. Kheder [5], Zainab M. Almarhoon [6] and Faiz A. Al-Aizari [6,7]

1. Department of Pharmaceutical Chemistry, College of Pharmacy, King Khalid University, Abha 61441, Saudi Arabia
2. Department of Physics, Faculty of Sciences, King Khalid University, P. O. Box 9004, Abha 61441, Saudi Arabia; halgarni@kku.edu.sa
3. Research Centre for Advanced Materials Science (RCAMS), King Khalid University, Abha 61441, 61413, P. O. Box 9004, Saudi Arabia
4. Department of Pharmacognosy, College of Pharmacy, King Khalid University, Abha 61441, Saudi Arabia; alsayari@kku.edu.sa (A.A.); ajmohsnah@kku.edu.sa (A.B.M.)
5. Department of Chemistry, Faculty of Science, Cairo University, Giza 12613, Egypt; nabila.abdelshafy@gmail.com
6. Department of Chemistry, College of Science, King Saud University, P.O. Box 2455, Riyadh 11451, Saudi Arabia; zalmarhoon@ksu.edu.sa (Z.M.A.); faizalaizari@yahoo.com (F.A.A.)
7. Department of Chemistry, Faculty of Science, AL-Baydha University, Albaydah 38018, Yemen

* Correspondence: ygaber@kku.edu.sa; Tel.: +966-17-241-9734

Method of antimicrobial evaluation of the synthesized thiazoline derivatives.

All microbial strains were provided from culture collection of the Regional Center for Mycology and Biotechnology (RCMB), Al-Azhar University, Cairo, Egypt. The antimicrobial activity was investigated on a newly synthesized compound in order to increase the selectivity of these derivatives towards test microorganisms using the agar diffusion method using Mueller-Hinton agar medium for bacteria and Sabouraud's agar medium for fungi. Briefly, 100 µl of the test bacteria/fungi were grown in 10 mL of fresh media until they reached a count of approximately108 cells/ml for bacteria or 105 cells/mL for fungi. All the newly synthesized compounds were weighed and dissolved in dimethyl sulfoxide to prepare extract stock solution. One hundred µL of each sample at 10 mg/mL was added to each well (10 mm diameter holes cut in the agar gel). The plates were incubated for 24-48 h at 37 °C (for bacteria and yeast) and for 48 h at 28 °C (for filamentous fungi). After incubation, the microorganism's growth was observed. The resulting inhibition zone diameters were measured in millimeters and used as criterion for the antimicrobial activity. The size of this clear zone is proportional to the inhibitory action of the compound under investigation. DMSO was used for dissolving the tested compounds thus used as solvent control and showed no inhibition zones, confirming that it has no influence on growth of the tested microorganisms. Positive controls were also performed using ampicillin and gentamycin as standard antibacterial drugs and *amphotericin B* as standard antifungal drug [53,54].

Method of evaluation of the cytotoxic effects of the synthesized compounds

Mammalian cell lines: HepG-2 cells (human Hepatocellular carcinoma) and HCT-116 (colon carcinoma) were obtained from the VACSERA Tissue Culture Unit. Chemicals Used: Dimethyl sulfoxide (DMSO), crystal violet and trypan blue dye were purchased from Sigma (St. Louis, MO, USA). Fetal bovine serum, DMEM, RPMI-1640, HEPES buffer solution, L-

glutamine, gentamycin and 0.25% trypsin-EDTA were purchased from Lonza (BioWhittaker®, Lonza, Belgium). Crystal violet stain (1%) is composed of 0.5% (w/v) crystal violet and 50% methanol made up to volume with ddH_2O and filtered through a Whatman No.1 filter paper.

Cell line Propagation:

The cells were propagated in Dulbecco's modified Eagle's medium (DMEM) supplemented with 10% heat-inactivated fetal bovine serum, 1% L-glutamine, HEPES buffer and 50μg/mL gentamycin. All cells were maintained at 37ºC in a humidified atmosphere with 5% CO**2** and were subcultured two times a week.

Cytotoxicity evaluation using viability assay: For cytotoxicity assay, the cells were seeded in 96-well plate at a cell concentration of 1×104 cells per well in 100 μL of growth medium. Fresh medium containing different concentrations of the test sample was added after 24 h of seeding. Serial two-fold dilutions of the tested chemical compound were added to confluent cell monolayers dispensed into 96-well, flat-bottomed microtiter plates (Falcon, Jersey, NJ, USA) using a multichannel pipette. The microtiter plates were incubated at 37ºC in a humidified incubator with 5% CO2 for a period of 24 h. Three wells were used for each concentration of the test sample. Control cells were incubated without test sample and with or without DMSO. The little percentage of DMSO present in the wells (maximal 0.1%) was found not to affect the experiment. After incubation of the cells at 37°C, for 24 h, the viable cells yield was determined by a colorimetric method [55].

In brief, after the end of the incubation period, media were aspirated and the crystal violet solution (1%) was added to each well for at least 30 minutes. The stain was removed and the plates were rinsed using tap water until all excess stain is removed. Glacial acetic acid (30%) was then added to all wells and mixed thoroughly, and then the absorbance of the plates were measured after gently shaken on Microplate reader (TECAN, Inc., San Diego, CA, USA), using a test wavelength of 490 nm. All results were corrected for background absorbance detected in wells without added stain. Treated samples were compared with the cell control in the absence of the tested compounds. All experiments were carried out in triplicate. The cell cytotoxic effect of each tested compound was calculated. The optical density was measured with the microplate reader (SunRise, TECAN, Inc.) to determine the number of viable cells and the percentage of viability was calculated as [(ODt/ODc)]x100% where ODt is the mean optical density of wells treated with the tested sample and ODc is the mean optical density of untreated cells. The relation between surviving cells and drug concentration is plotted to get the survival curve of each tumor cell line after treatment with the specified compound. The 50% inhibitory concentration (IC_{50}), the concentration required to cause toxic effects in 50% of intact cells, was estimated from graphic plots of the dose response curve for each conc. using the GraphPad Prism software (GraphPad Inc., San Diego, CA. USA).

X-ray Analysis

The thiazole derivative **5c** was obtained as single crystals by slow evaporation from ethanol solution of the pure compound at room temperature. Data were collected on a Bruker APEX-II D8 Venture area diffractometer, equipped with graphite monochromatic Mo $K\alpha$ radiation, λ = 0.71073 Å at 293 (2) K. Cell refinement and data reduction were done by Bruker SAINT. Solving the thiazole structure was carried out using SHELXT [56,57]. The final refinement was done by full-matrix least-squares techniques with anisotropic thermal data for nonhydrogen atoms on F. CCDC 1818811 contains the supplementary crystallographic data for this compound can be obtained free of charge from the Cambridge Crystallographic Data Centre via www.ccdc.cam.ac.uk/data_request/cif.

i

1-(4-Methyl-3-phenyl-2-thioxo-2, 3-dihydrothiazol-5-yl) ethan-1-one.(2a)

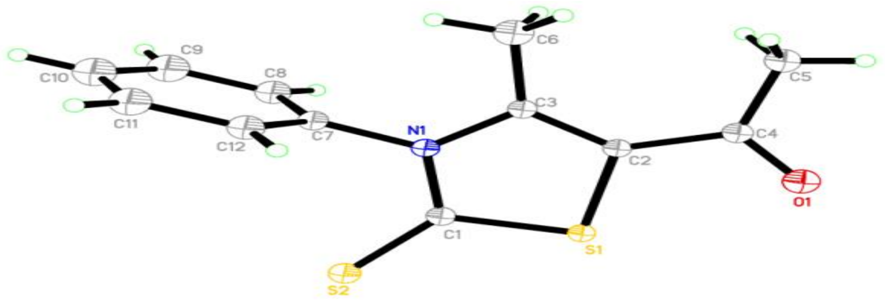

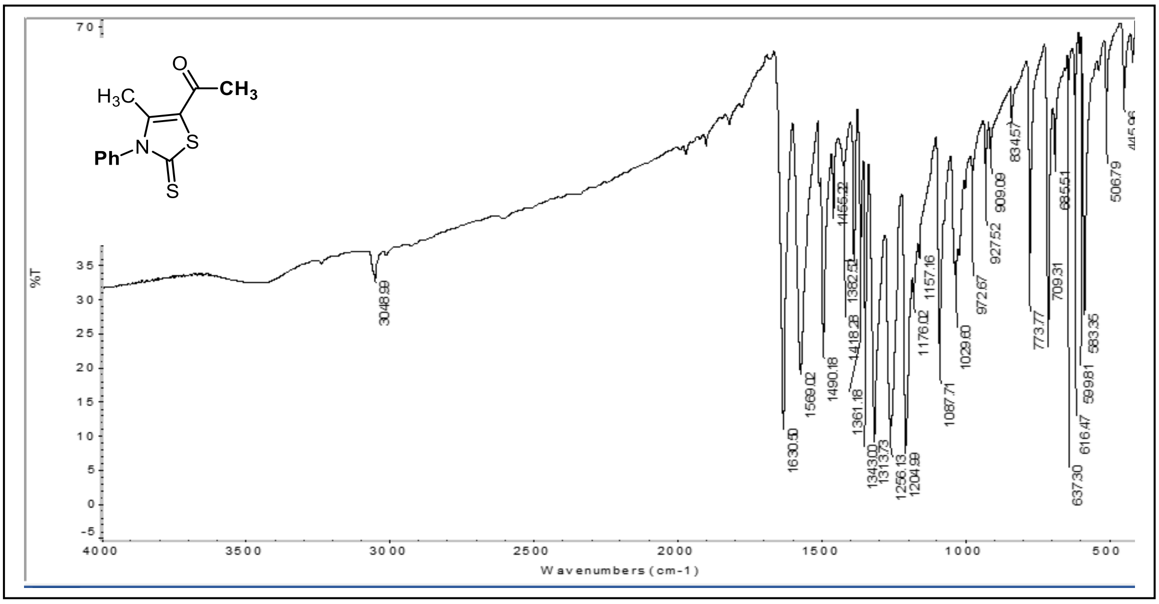

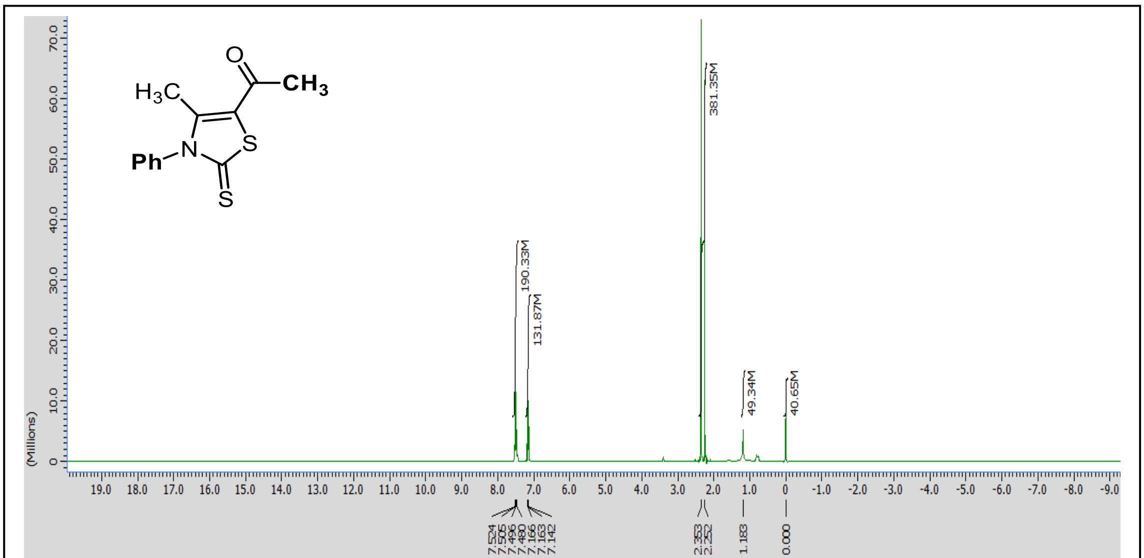

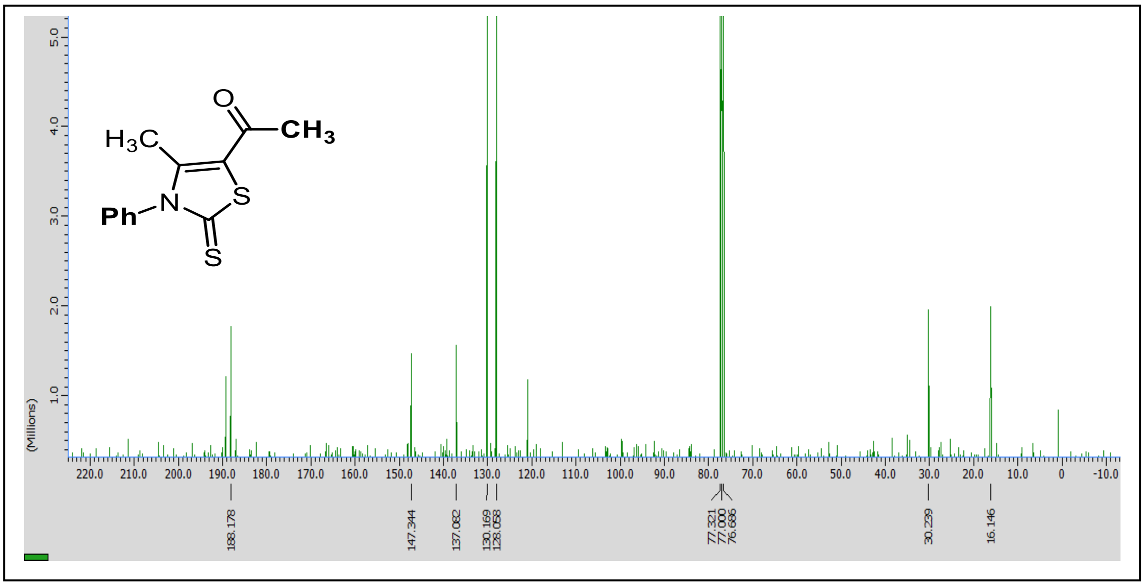

Ethyl 4-methyl-3-phenyl-2-thioxo-2,3-dihydrothiazole-5-carboxylate (2b)

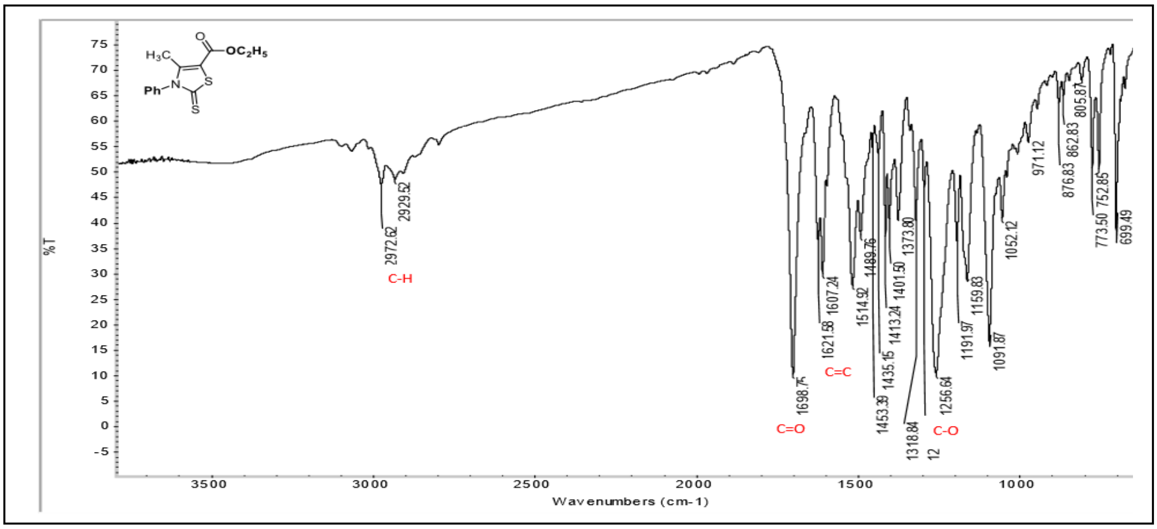

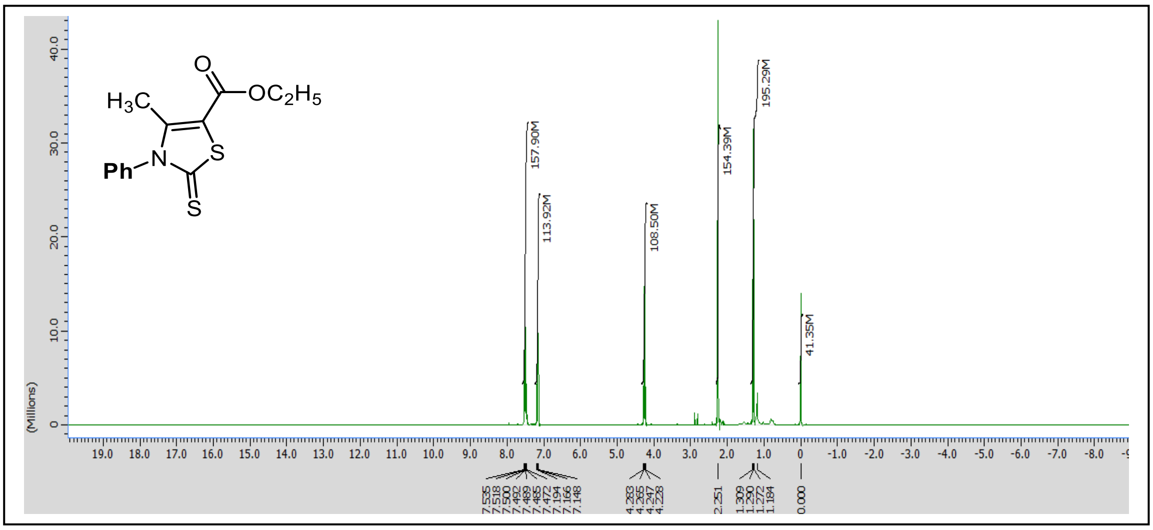

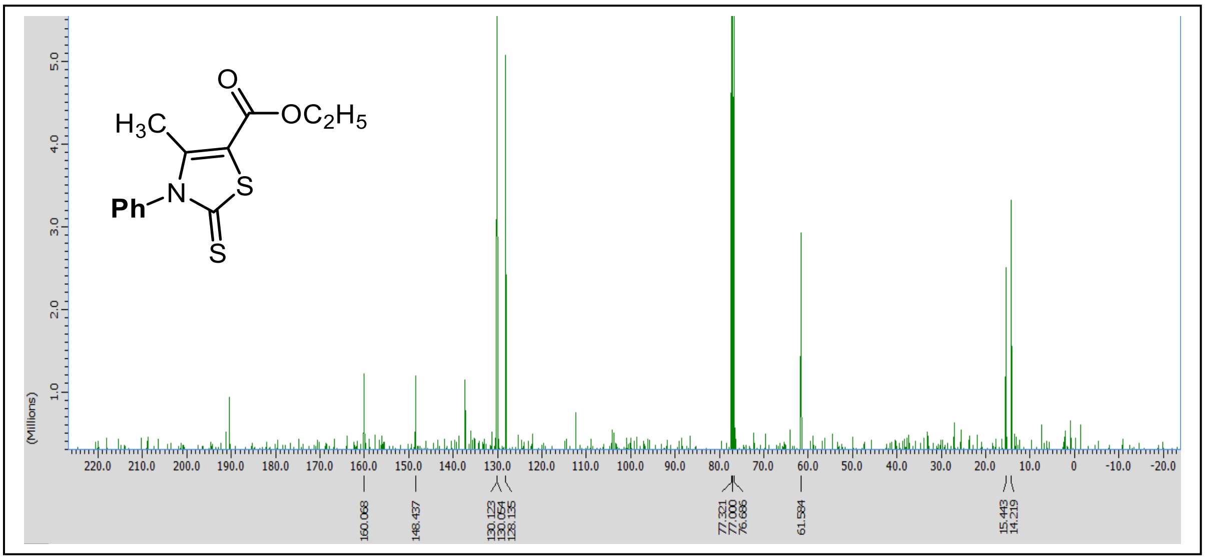

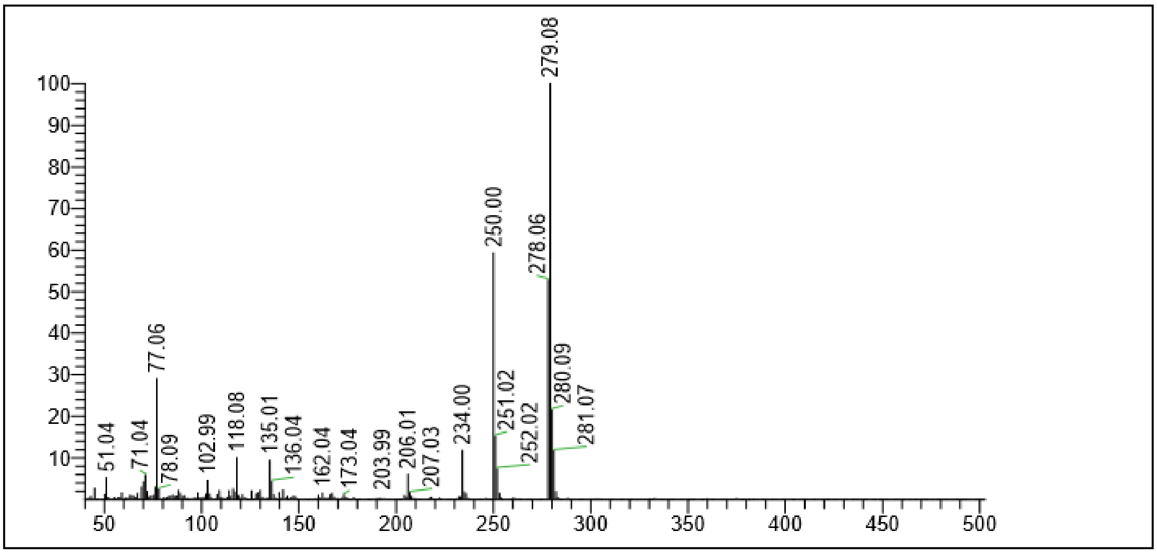

1 -(4-Methyl-2-thioxo-2,3-dihydrothiazol-5-yl)ethan-1-one. (2c)

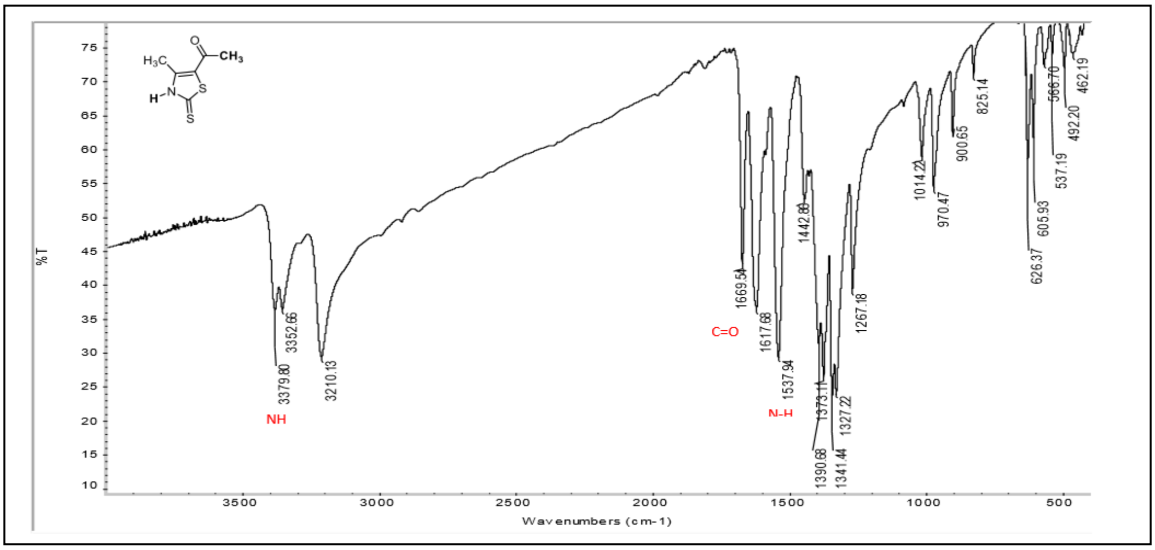

iv

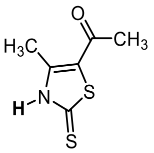

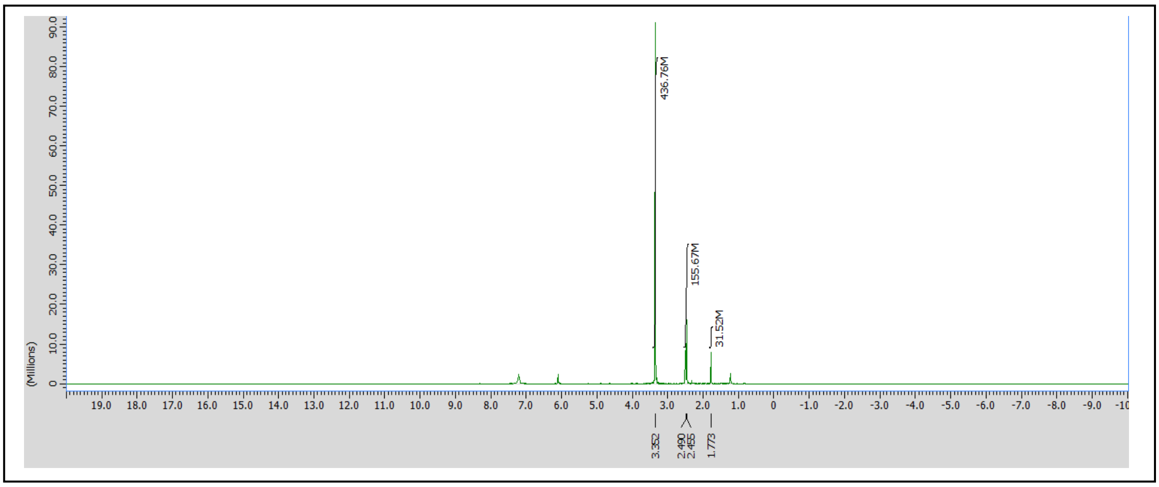

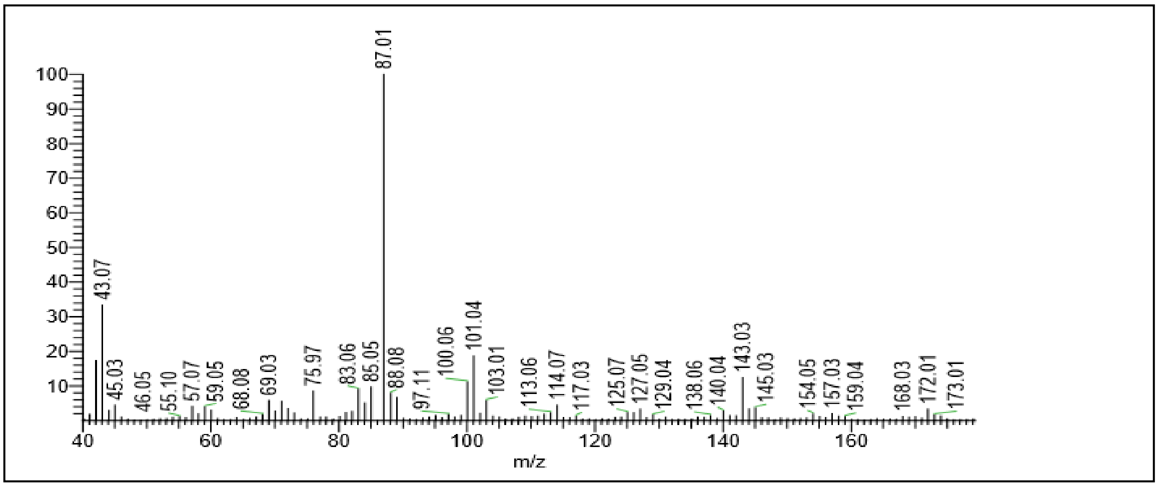

Ethyl 4-methyl-2-thioxo-2,3-dihydrothiazole-5-carboxylate (2d):

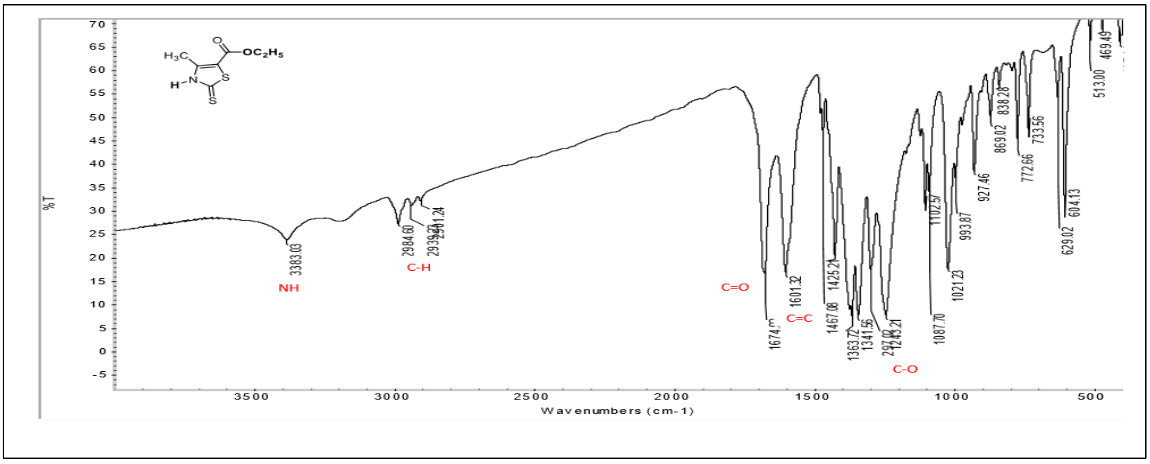

V

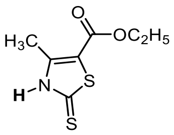
O
H3C
OC2H5
H
N
S
S

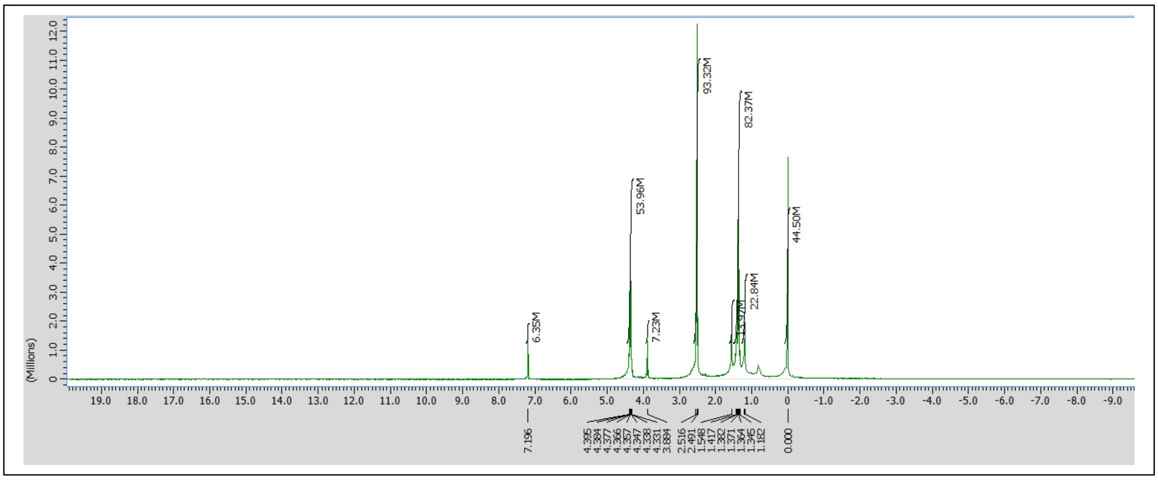
(Millions)
6.35M
53.96M
7.23M
93.32M
82.37M
13.47M
22.84M
44.50M
7.196
4.395
4.384
4.377
4.366
4.357
4.347
4.338
4.331
3.884
2.516
2.491
1.548
1.417
1.382
1.371
1.364
1.345
1.182
0.000

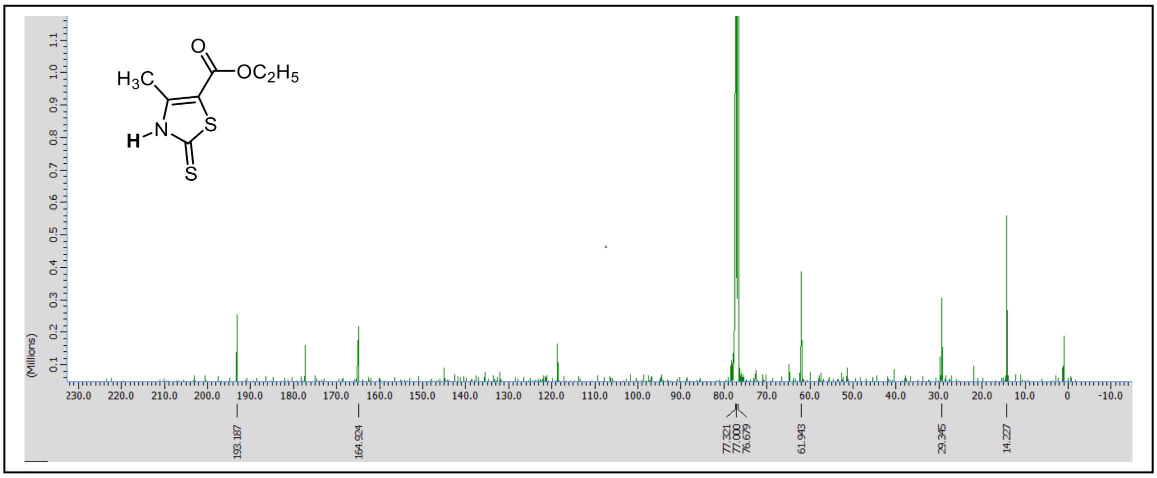
O
H3C
OC2H5
H
N
S
S
(Millions)
193.187
164.924
77.321
77.000
76.679
61.943
29.345
14.227

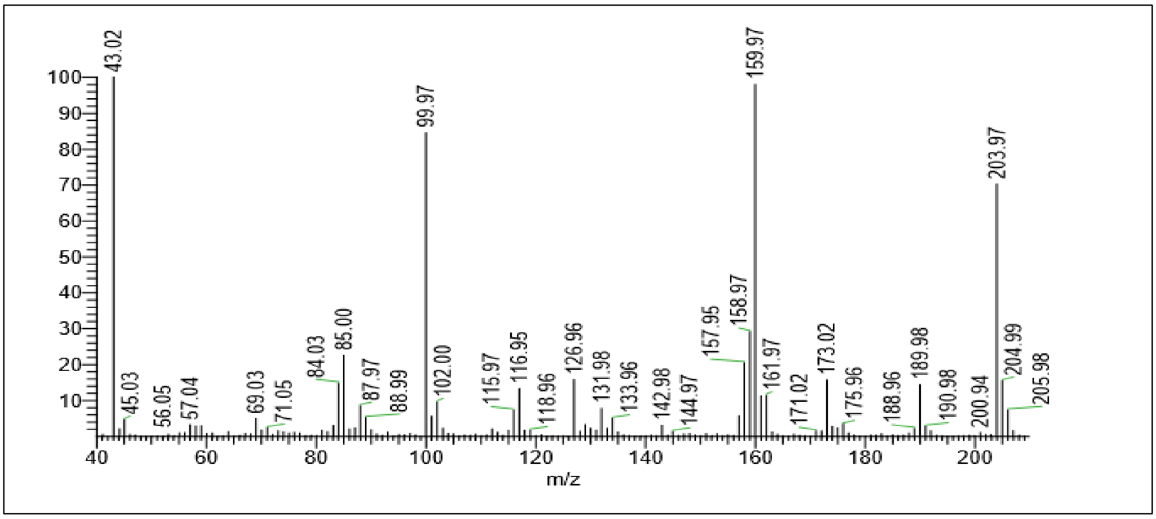
43.02
45.03
56.05
57.04
69.03
71.05
84.03
85.00
87.97
88.99
99.97
102.00
115.97
116.95
118.96
126.96
131.98
133.96
142.98
144.97
157.95
158.97
159.97
161.97
171.02
173.02
175.96
188.96
189.98
190.98
200.94
203.97
204.99
205.98
m/z

Ethyl 4-methyl-3-(phenylamino)-2-thioxo-2,3-dihydrothiazole-5-carboxylate (2e

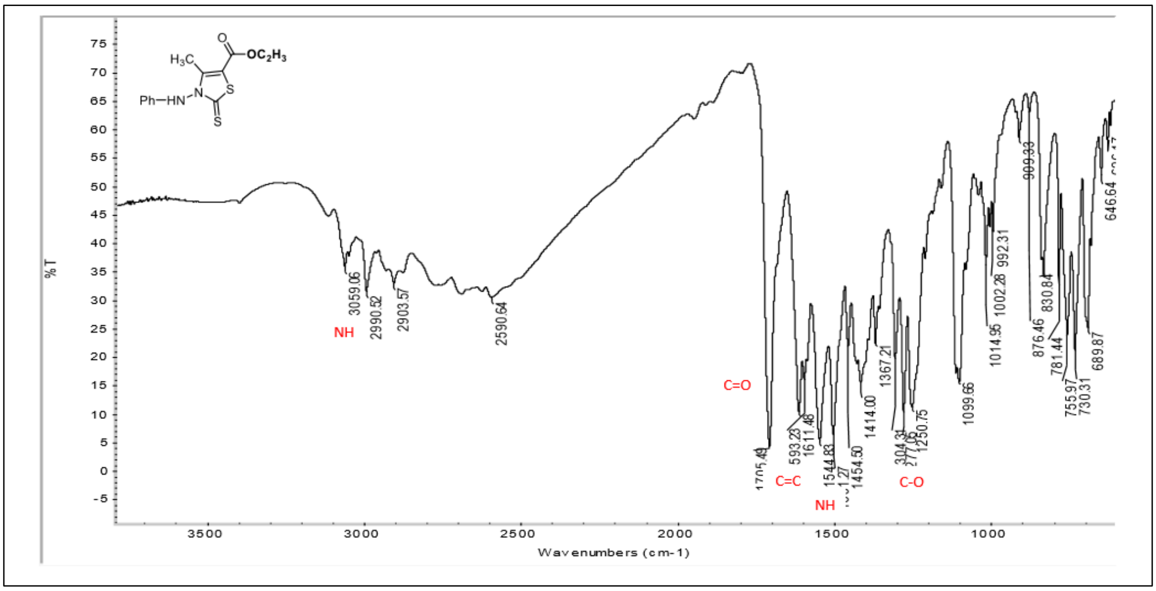

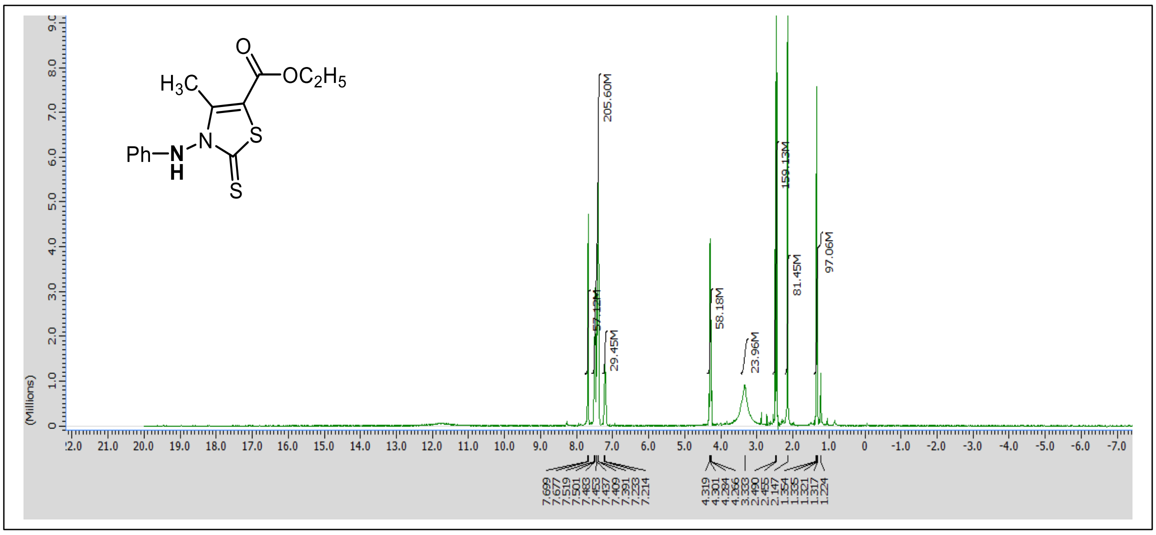

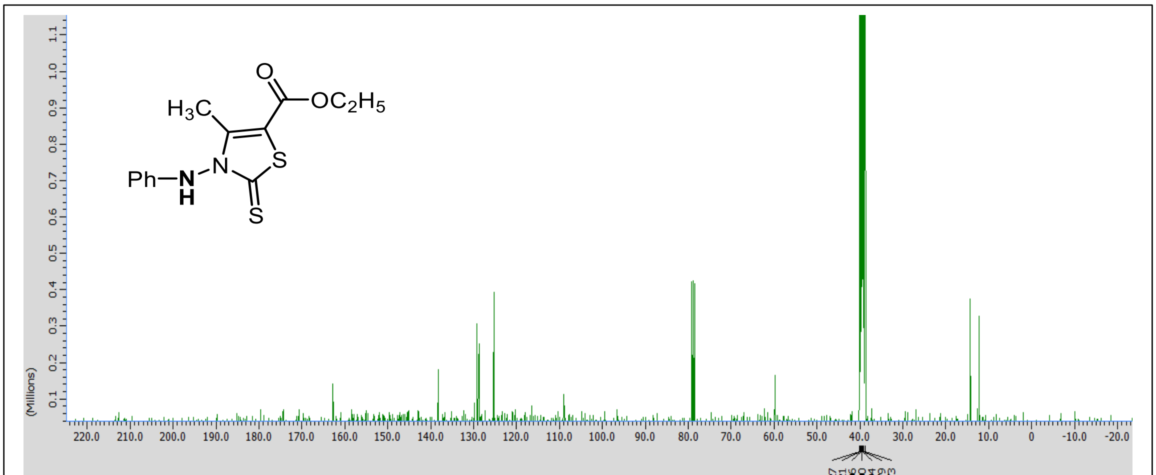

vii

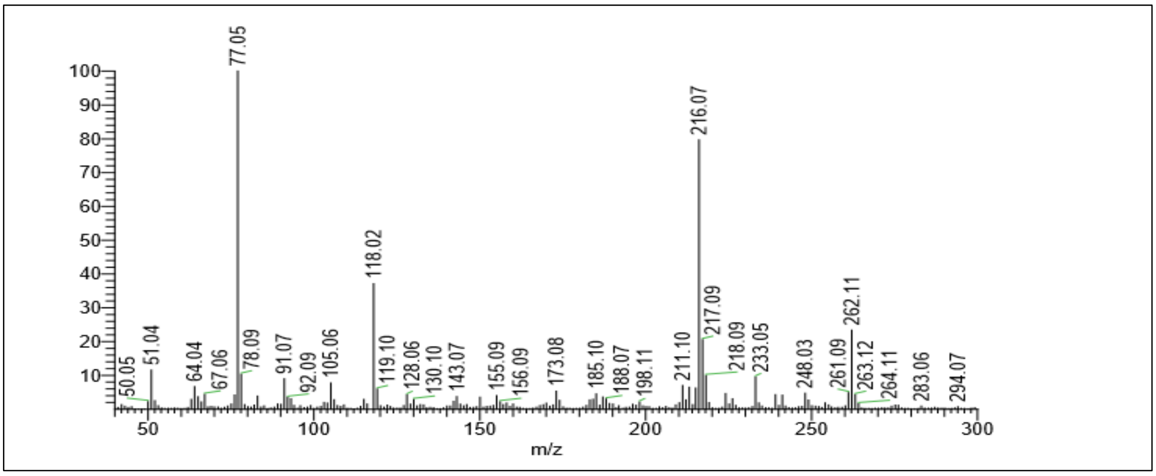

100
90
80
70
60
50
40
30
20
10
50.05
51.04
64.04
67.06
77.05
78.09
91.07
92.09
105.06
118.02
119.10
128.06
130.10
143.07
155.09
156.09
173.08
185.10
188.07
198.11
211.10
216.07
217.09
218.09
233.05
248.03
261.09
262.11
263.12
264.11
283.06
294.07
50
100
150
200
250
300
m/z

(*E*)-4-methyl-3-phenyl-5-(1-(2-phenylhydrazono) ethyl)thiazole-2(3*H*)-

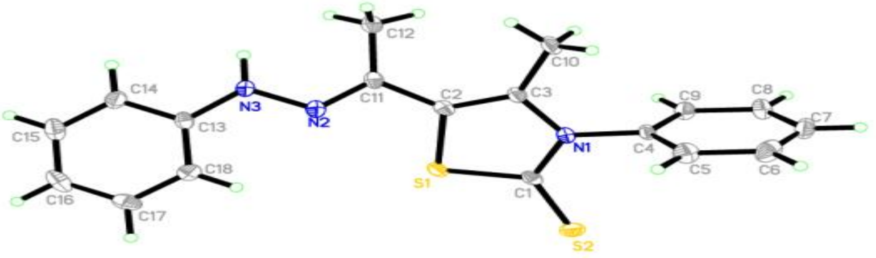

thione (5c)

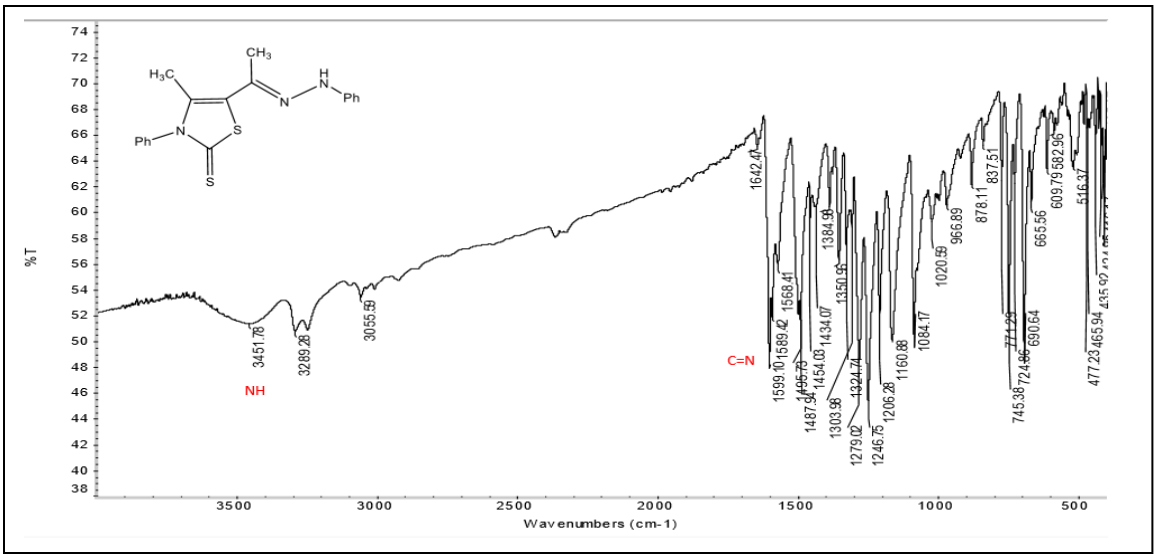

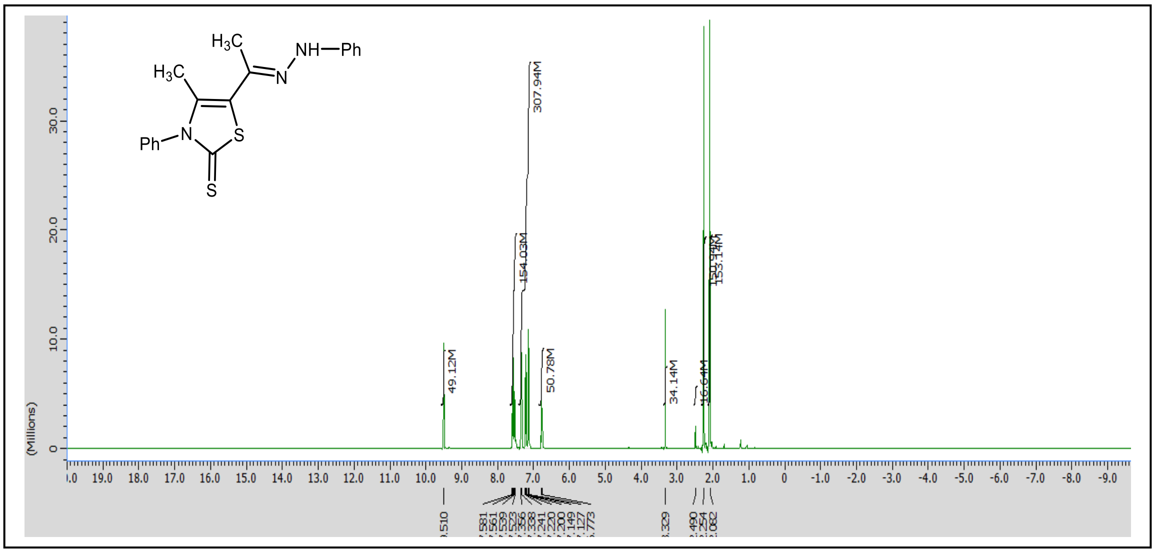

ix

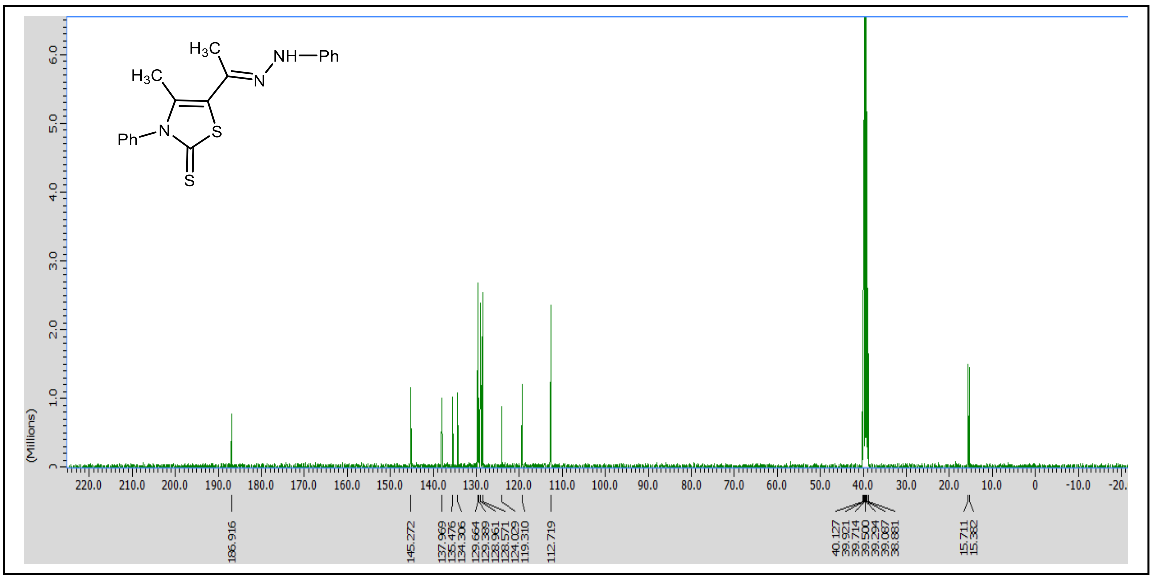
H3C
NH—Ph
N
H3C
Ph—N
S
S
(Millions)
220.0 210.0 200.0 190.0 180.0 170.0 160.0 150.0 140.0 130.0 120.0 110.0 100.0 90.0 80.0 70.0 60.0 50.0 40.0 30.0 20.0 10.0 0 -10.0
186.916
145.272
137.969
135.476
134.306
129.664
129.389
128.961
128.571
124.029
119.310
112.719
40.127
39.921
39.714
39.500
39.294
39.087
38.881
15.711
15.382

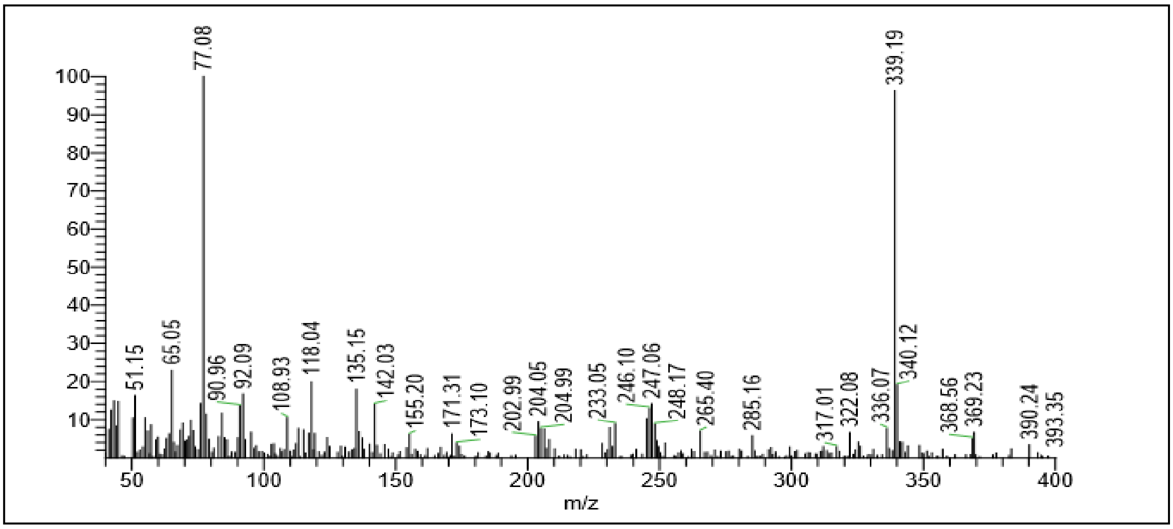
77.08
339.19
340.12
51.15
65.05
90.96
92.09
108.93
118.04
135.15
142.03
155.20
171.31
173.10
202.99
204.05
204.99
233.05
246.10
247.06
248.17
265.40
285.16
317.01
322.08
336.07
368.56
369.23
390.24
393.35
m/z
